# A comparison of doctoral training in biomedicine and medicine for some UK and Scandinavian graduate programmes: learning from each other

**DOI:** 10.1002/2211-5463.12629

**Published:** 2019-03-30

**Authors:** Anwen Williams, Meriel G. Jones, Roland Jonsson, Robert A. Harris, Michael J. Mulvany

**Affiliations:** ^1^ School of Medicine Cardiff University UK; ^2^ Institute of Integrative Biology University of Liverpool UK; ^3^ Faculty of Medicine University of Bergen Norway; ^4^ Department of Clinical Neuroscience Karolinska Sjukhuset Karolinska Institutet Stockholm Sweden; ^5^ Department of Biomedicine Aarhus University Aarhus Denmark

**Keywords:** PhD admission, PhD outcomes, PhD supervision, PhD thesis, PhD training

## Abstract

Although the historical bases for graduate training in the United Kingdom (UK) and Scandinavia both stem from the original concept developed by von Humboldt, and both award a ‘PhD degree', their paths have diverged. There are thus significant differences in the manner in which graduate training is organised. To analyse these differences, two UK graduate programmes (School of Medicine, Cardiff University; Institute of Integrative Biology, University of Liverpool) and two Scandinavian graduate schools (Faculty of Medicine and Dentistry, University of Bergen; Karolinska Institutet, Stockholm) completed a Self‐evaluation questionnaire developed by Organisation of PhD Education in Biomedicine and Health Sciences in the European System (ORPHEUS)). Analysis of the completed questionnaires shows differences concerning requirements for admission, the training content of PhD programmes, the format of the PhD thesis, how the thesis is assessed and the financial model. All programmes recognise that PhD training should prepare for employment both inside and outside of academia, with emphasis on transferable skills training. However, the analysis reveals some fundamental differences in the direction of graduate programmes in the UK and Scandinavia. In the UK, graduate programmes are directed primarily towards teaching PhD students to do research, with considerable focus on practical techniques. In Scandinavia, the focus is on managing projects and publishing papers. To some extent, the differences lead to a lack of full recognition of each other's theses as a basis for doing a postdoc. This paper describes the basis for these differences and compares the two approaches and points to areas in which there is, or might be, convergence.

**Abbreviations**
‘Bergen': Faculty of Medicine, University of Bergen
‘Cardiff': School of Medicine, Cardiff University
‘Karolinska': Karolinska Institutet, Stockholm
‘Liverpool': Institute of Integrative Biology, University of Liverpool
ORPHEUS: Organisation of PhD Education in Biomedicine and Health Sciences in the European System
UK: United Kingdom

There are a number of substantial differences between PhD programmes in the United Kingdom (UK) and Scandinavia. These can be traced to differences in the manner in which doctoral training has developed in the two regions since the original concept of a PhD (dr. phil.) was developed by Wilhelm von Humboldt in 1810. He recognised that research required professional training and introduced programmes whereby PhD students did research under supervision and completed their studies by defending a thesis. This concept spread to other European countries, including the Netherlands and France 1. Although the concept spread to the United States, and Yale gave the first PhD in 1861, the idea of giving a doctorate for research training did not reach the UK before 1917, when Oxford instituted its first research doctorate programme (DPhil). With its introduction, the university was anxious to clarify that the DPhil was at a lower level than other doctorates that were awarded at that time, for example DSc and DLitt, which were based on the academic having a portfolio of published research of a particularly high standard 1. In Scandinavia, doctoral programmes have until comparatively recently also been awarded to those with a high standard of published research. More recently, programmes associated with research training were restructured as PhD programmes (1981 Sweden, 1990 Denmark, 2003 Norway) but the tradition of these being based on published research was maintained. Furthermore, with this change the Scandinavian governments recognised that PhD training should be aimed at filling not only academic positions, but also employment positions in the wider job market. Appropriate regulations were introduced, and funding was made available to ensure that PhD training developed competences that would be of use to society as a whole.

Another important difference between the UK and Scandinavia relates to what constitutes ‘undergraduate' education. In the UK (at least in England and Wales), this refers traditionally to completion of a 3‐year bachelor degree programme, whereas in Scandinavia, it refers to a 5‐year ‘candidate education'. Consistent with the Bologna process 2, candidate education is now split between bachelor and master's programmes, but in practice a Scandinavian master's programme is a continuum of a bachelor programme. Although as discussed below, integrated master's programmes are increasingly being introduced in the UK, the traditional basis for admission to a PhD programme differs between the UK and Scandinavia: in UK, admission requires a completed bachelor programme, whereas in Scandinavia it requires a completed bachelor‐plus‐master's programme, where the master's programme will normally have included a substantial (1‐year) research project.

A third important difference is the business model. In the UK, there are substantial tuition fees for conducting a PhD and although many PhD students receive stipends, many do not. In Scandinavia, all PhD students have salaries and there are no tuition fees.

The fourth, and perhaps most fundamental difference, is the nature of the thesis. In UK, consistent with the original von Humboldt concept, the primary outcome is a lengthy thesis (up to 250–300 pages) that describes the work that the student has done, the relation of the results to the existing literature, and the perspectives. In Scandinavia, the thesis consists of papers (or manuscripts) arising from the work and a summary that covers the same points as the UK thesis but much shorter (typically around 50 pages). While students are responsible for all the work done, the students need not necessarily have done all the work themselves (something that would be anathema in the UK!).

It should be noted that PhD students are known as PhD candidates in Norway, but as PhD students in UK and Sweden. In this paper, the term PhD student is used, even though there is a broad wish from many PhD students that they should be termed ‘candidates' 3 or the increasingly common title ‘early career researcher'.

As discussed below, these differences in background have led to significant differences in the approaches to PhD programmes in the UK and Scandinavia. In the UK, the PhD is considered an educational continuation of a bachelor programme, giving relatively young PhD students the opportunity to learn about research under expert supervision. The work should provide publishable material, but publication itself is not seen as a primary aim and the arguments for tuition fees are persuasive. In Scandinavia, by contrast, the somewhat older and longer educated PhD students will most often already have had research experience. In Scandinavia, supervisors expect their PhD students to support their research group's activities with publications and the salary and the PhD degree they receive is recompense for this. The Scandinavian approach is also followed to a large extent by other European countries 4.

Given these different backgrounds, both the UK and Scandinavian approaches are understandable. But since both approaches lead to an equivalent ‘PhD degree' title, it is natural to enquire whether the competences of PhD graduates are similar in the two regions. While both the UK and Scandinavia will strongly defend the basis for their own programmes, sceptical views are sometimes heard about the other's approach as indicated in Table 1. It is the purpose of this paper to examine the differences in approaches in the hope that both regions may learn from each other.

**Table 1:** Some myths and prejudices about PhD programmes in UK and Scandinavia

UK view of Scandinavian PhD	Scandinavian view of UK PhD
PhD students may use technicians to perform their experiments and do not get hands‐on research experience	PhD students play an important role as technicians, perhaps at the expense of responsibility for the complete project
The oral defences are set‐pieces, and the PhD thesis is not examined with the necessary rigour	Lack of transparency in the closed defence. No need to demonstrate ability to defend work publicly
Since published papers usually have several authors, it is hard to determine the contribution of the PhD student	Lack of published papers makes it difficult to assess the merits of a PhD graduate
Supervisors want their PhD students to publish and may not be so interested in the training aspect	Lack of 2‐year master's requirement for entry may reduce the quality of a UK PhD compared to Scandinavia

## Material and methods

To examine the differences in approach of UK and Scandinavian PhD programmes, it was decided to make a detailed comparison of four bioscience, biomedical and medical PhD programmes, two in the UK and two in Scandinavia. The programmes concerned (School of Medicine, Cardiff University; Institute of Integrative Biology, University of Liverpool; Faculty of Medicine, University of Bergen; Karolinska Institutet, Stockholm) are considered to be typical, and although it is recognised that graduate programmes are not identical within the two regions, we believe that the data provide an indication of the situation in each region. For brevity, the programmes are referred to in this paper as ‘Cardiff', ‘Liverpool', ‘Bergen' and ‘Karolinska'. The programme at Karolinska includes is termed Medical Science and includes medicine, dentistry and biosciences.

To make this comparison, we have used the ORPHEUS Self‐evaluation questionnaire 5. This is based on 68 recommendations developed by ORPHEUS (Association of Medical Schools in Europe and World Federation for Medical Education to describe best practices in PhD training 6, with input of institutions from almost all countries in Europe. The recommendations are concerned with the research environment, outcomes, admission policy and criteria, PhD training programme, supervision, PhD thesis, assessment and graduate school structure. For each area, there are basic recommendations, which it is suggested that all graduate schools should fulfil, and ‘quality development' recommendations that are in accordance with international consensus about good practice. The recommendations describe not only the aims but also the content of PhD programmes. In addition, there are annotations that clarify terms and indicate flexibility. A similar comparison has been made recently between US and European graduate schools, where the data from Karolinska Institutet were also included 7.

To provide a basis for comparing the four graduate programmes, each programme completed the Self‐evaluation questionnaire by providing a description of how their programme deals with each recommendation. For example, there is a recommendation (BR1.4) that ‘There should be arrangements to allow PhD candidates [students], if relevant, to perform part of their PhD programme at another institution, including those in other countries'. Here the Self‐evaluation questionnaire asks institutions to ‘Describe the arrangements provided for allowing PhD candidates [students] to spend part of their time in another institution. How many take advantage of these arrangements? Who covers the expenses?'

The questionnaires have been completed by the heads of the graduate programmes concerned in consultation with other stakeholders. The responses to each of the 68 points in the questionnaire (denoted lines #1–#68) are compared in Table S1 where differences are highlighted (i.e. given grades 1 or 2). The full responses from each of the four graduate programmes are listed side by side in Table S2.

## Results

### Research environment

See Table S1, lines #1–6. All the graduate programmes included in this investigation are based in universities that are among the top in their respective countries, and thus, all have facilities for completing PhD projects. High ethical standards are maintained. All graduate programmes collaborate with other graduate programmes and possibilities for joint PhD degrees are either available (Scandinavia) or being worked on (UK). PhD students have the possibility to do part of their PhD studies at another graduate programme, although relatively few take advantage of this, largely due to time constraints imposed by having only around 2&frac12; years to do the research project.

### Outcomes

See Table S1, lines #7–9. All graduate programmes recognise the need for PhD students to develop competences for employment either within or outside of academia. At Cardiff and Liverpool, there are formal arrangements to monitor needs analysis and development. At Bergen and Karolinska, it is more often left to the PhD student and the supervisor to decide what is needed, but PhD students are required to take a substantial number of courses in transferable skills to ensure that they have the necessary competences. The need for career assistance is widely recognised. At Cardiff, there is career assistance at induction and this continues throughout the programme. At Liverpool, assistance with career assistance is available. At Bergen, PhD students are encouraged to make career plans at induction, courses are available, and there is an annual Career Day. At Karolinska, there is a central career service that organises professional development courses available to all PhD students, career days and subject‐specific seminars. It is also the remit of Karolinska supervisors to discuss career planning with their PhD students, and the first part of the annual assessment form specifically addresses this aspect.

None of the graduate programmes has formal mechanisms for providing all PhD students with career advice, although supervisors have this as a nominal task.

### Admission policy and criteria

See Table S1, lines #10–16. In cases where the graduate programmes or other funding bodies are offering stipends, the application and selection process is transparent and competitive. Where there is alternative funding (e.g. clinical PhD students employed as practicing doctors, industrial PhD students), applications may be accepted without (direct) competition following evaluation of the PhD student's ability and the quality of the project. All graduate programmes require approval of the project by an independent panel prior to admission and require that a plan for financing of the PhD programme is in place before admission. All graduate programmes may take account of previous research experience although such experience is not an absolute requirement. In Scandinavia, such experience will normally be part of their master's degree. All graduate programmes allow extra time if the PhD student has other employment during their PhD.

As mentioned above, a major difference concerns the educational requirement for admission to a PhD programme (Table S1 line #11). At Cardiff and Liverpool, applicants should have a bachelor's degree (first or upper second) or lower second and master's degree; those who have had additional research experience (e.g. through summer project work) will have a competitive advantage in the application process. In contrast, at Bergen and Karolinska, applicants should have a 5‐year master's degree (that includes a 6‐ to 12‐month research project) or a medical or other professional degree, where again previous research experience will increase chances of acceptance to a programme.

### PhD training programme

Table S1, lines #17–25. All of the graduate programmes are based on original research and have annual assessments of progress. It is expected that this, together with, for example journal clubs, participation in national and international meetings, and manuscript writing will provide training in analytical and critical thinking. A course in ethics is mandatory for all graduate programmes. The nominal length of the PhD programme (including time to write up the thesis) is 3.5–4 years in Cardiff and Liverpool and 4 years at Karolinska. At Bergen, the stipulated 3 years for a PhD programme may be extended to 4 years if PhD students have teaching duties. For all programmes, extensions are possible.

Specific arrangements are in place for all graduate programmes to allow PhD students to conduct their PhD programme in parallel with clinical studies, for example 50% research and 50% clinic. At Bergen, medical students have the possibility to do a MD‐PhD where PhD studies are integrated with the MD programme, somewhat similar to the MB‐PhD programmes found at some UK universities 8.

An important difference concerns the provision of courses and the requirement to follow them (Table S1 line #21). At Cardiff and Liverpool, training courses are available but are not a compulsory part of a PhD programme. At Bergen and Karolinska, a comprehensive programme of training courses is available, and course participation/educational activities corresponding to 6 months (30 ECTS, ca. 60 UK credits) are required for all PhD students.

### Supervision

Table S1, lines #26–39. All graduate programmes have structured supervision with one principal supervisor and one or more cosupervisors. For all graduate programmes, supervisors must have a doctoral degree or equivalent academic competence within the subject area, and be an active researcher. For all graduate programmes, a solid publication record is taken to imply that supervisors have scientific networks.

Before admission, at Liverpool, Bergen and Karolinska, supervisors and PhD students are matched by mutual consent. At Cardiff, matchmaking often is based on the student having an interest in a particular project.

At Cardiff, a supervisor may have up to six PhD students. At Bergen, Liverpool and Karolinska, there is no formal limit. In practice, three is the norm for all graduate programmes. For all graduate programmes, supervisors have regular meetings with their PhD students, although the frequency is not strictly defined (Cardiff: ‘in accordance with an agreed frequency'; Liverpool: ‘minimum requirement of once per month for full‐time PhD students and once per 2 months for part‐time. In practice, most supervisors have “open door” policy and regular group meetings so meet much more frequently'; Bergen: ‘expected to meet several times per month'; Karolinska: ‘daily, weekly or monthly physical meetings'). All graduate programmes recognise that conflicts sometimes arise and have procedures for helping to resolve conflicts.

Regarding supervisor training (line #30), Cardiff and Karolinska have capacity for training large numbers of supervisors. At Cardiff, 226 academics have received supervisor training over the past 2 years. Karolinska has a 1‐week ‘basic' training course that is compulsory for all new supervisors (281 supervisors have attended basic courses over the past 2 years; 96 supervisors have attended advanced courses during the same period). Other courses are available. At Bergen, there is a compulsory e‐learning course for all principle supervisors and an annual 1‐day seminar for all supervisors. In addition, a supervisor training course is available and there are regular 2‐h supervision seminars. At Liverpool, supervisor training courses are available, but at present more limited.

For the UK graduate programmes, PhD students will usually have a ‘mentor' who can give general advice independently of the supervisor. This is also the case at Karolinska, but at Bergen this is not yet the practice (line #39).

### PhD thesis

Table S1, line #40–49. For all graduate programmes, English is the norm and by far the most common for both the thesis and the examination/defence. At Cardiff, Welsh is allowed. At Bergen and Karolinska, Scandinavian languages are allowed.

As described in the Introduction, there are major differences in the format of the thesis for the UK and Scandinavian graduate programmes (line #41). At Cardiff and Liverpool, the thesis is usually in the form of a 250–300 page bound monograph (Liverpool: ‘no more than 100 000 words') that provides the background for the project, the methodology and the results together with a discussion and perspectives. Papers may be included as chapters. At Karolinska, and particularly Bergen, the emphasis is on published/accepted papers and submitted manuscripts, preferably in journals with high impact factor. A thesis will normally contain 2–3 papers/manuscripts. In addition, there is a summary providing a review of the literature, critical assessment of the methods and discussion of the results. This summary will normally have a length of around 50 pages, plus the accompanying papers or manuscripts. Lay summaries of the thesis may be published in Scandinavia but this is not usual in the UK (line #49).

### PhD thesis assessment

Table S1, line #50–59. For all programmes, students may only be allowed to defend their thesis on the recommendation of their supervisor. Furthermore, at Bergen, students are required to give a 45‐min lecture before a panel on a specified topic; only if this is satisfactory will the student be allowed to proceed to the public defence. There are, however, significant differences in the manner in which PhD theses are assessed.

Consistent with UK tradition, at Cardiff and Liverpool the thesis is examined in a closed session *viva voce* by two independent examiners, usually but not always chosen from within the UK (the key requirement is understanding of the UK examination procedure). In contrast, following Scandinavian tradition, the thesis at Bergen and Karolinska is publicly defended. At Bergen, two independent opponents (usually one from abroad) and a chairman first assess the thesis with a written evaluation of about five pages and are subsequently opponents during the public defence. At Karolinska, the thesis and the public defence are assessed by an Examination Board of at least three experts, one of whom is from another (Swedish) graduate programme. The defence consists of a discussion between the PhD student and an invited ‘faculty opponent' who is usually external and often from another country. At both Bergen and Karolinska, questions can be posed by the attending audience.

For the UK graduate programmes, there are progress monitoring procedures, with a training needs analysis every 6 months. At Bergen and Karolinska, PhD students will have completed courses in transferable skills and will have had some form of examination in these. While the assessment committees do not for any of the graduate programmes normally make a formal assessment of transferable skills competences (apart from assessment of the lecture that forms part of the defence), at Karolinska there is training for faculty to learn how to pose questions that assess these and other intended learning outcomes. A portfolio listing courses taken is appended to the thesis diploma at Bergen, while Cardiff and Liverpool have facilities that allow PhD students to record their portfolio. At Karolinska, a personal portfolio will soon supplement the current official transcript record.

### Graduate school structure

Table S1, line #60–68. Graduate training for all of the graduate programmes is organised through a consolidated administration covering all PhD students in the fields concerned (at Liverpool this in the process of being established). At Cardiff and Liverpool, PhD student progress is monitored by independent panels. At Bergen and Karolinska, formal reports are submitted to the administration annually; both graduate programmes have independent review committees that assess progress during the project half‐way through the programme.

All graduate programmes have websites that describe the programmes and provide PhD students with the information they need.

### PhD students

PhD students for all graduate programmes have representation in the various committees and boards responsible for PhD training. Confidential counselling for PhD students is available for all graduate programmes, although the arrangements differ (Table S1 line #67).

There are significant differences in the financial arrangements for PhD students. In UK, there are annual tuition fees amounting to ca. €5000 for UK and EU PhD students and ca. €22 000 for PhD students from other countries; there may also be bench fees. Most UK and EU PhD students receive tax‐free stipends to cover living expenses, and tuition and bench fees, while others have to obtain alternative financial support including income from paid employment. At Bergen and Karolinska, there are no tuition or bench fees and students are employed as junior staff with full employment benefits.

### Outcomes

At Karolinska, approximately half of all PhD students are clinicians or other healthcare professionals, and following graduation continues with their employment. The other PhD graduates proceed primarily to postdoc positions or join biotechnology companies. An investigation of alumni graduating in 2010 and 2014 revealed that all graduates considered that their PhD training was useful in their current employment, and all were working (except a few who were on sick leave). In Norway, the NIFU have determined the employment of PhD graduates 9, finding that 90% are in relevant jobs (primarily in the public sector, but 25% in the private sector). Of those in medicine and health sciences, 53% are in R & D and 31% in clinical work. At Liverpool, around 80% of PhD graduates in employment enter research careers in the academic, public or private sectors. At Cardiff, a study showed 58% remaining in Higher Education settings with the majority of the remainder embarking on science careers outside research or nonscience sector occupations.

## Discussion

The PhD is an internationally recognised degree despite the differences in approach 10, 11. However, given the major scope of and investment in graduate training across the world there is substantial discussion about the wisdom of this effort 12, 13. This debate is highlighted by the realisation that only a fraction of PhD graduates will in the long run continue to use their talents in academia or even in dedicated research activities 14, 15, 16.

### The convergence of doctoral training timelines

Although the nominal time span from starting bachelor to completing PhD training varies from 6 years in the UK to 8 years in Norway and 9 years in Sweden, there is some convergence. In the UK, 4‐year bachelor programmes including a substantial research component are becoming more common, while PhD programmes are often extended to 4 years. Thus, for the UK programmes, the total time from starting bachelor to completing PhD for most students is now around 7–8 years, dependent on whether the student has followed a master's. At Bergen, the current overall time of 8 years from starting bachelor to completing PhD is often extended to 9 years with PhD students taking on teaching duties (about 8 h per week) during the training period. At Karolinska, the average net PhD graduation time is currently 4.5 equivalent years, thus 9.5 years from starting bachelor.

### PhD programme

As indicated in the Results section, PhD programmes in the UK are directed primarily towards teaching PhD students to do research. PhD students will thus normally do all the work with little assistance from technicians. The courses in transferable skills that are provided recognises that PhD graduates will seek employment not only in academia but outside of it.

In Scandinavia, there appears to be a greater emphasis (at least as seen by the governments that fund the programmes) on PhD training producing persons who are able to contribute to the knowledge society. Thus, PhD students should not only learn to conduct high‐quality research, but should also produce and publish results. There is also considerable emphasis on PhD students developing a range of transferable skills including communication skills with the requirement that all PhD students should follow a course programme (ca. 6 months). The techniques of research methodology are learnt at master's level, and there is acceptance that much of the research is collaborative, and that some of the laboratory work may be performed by technicians.

### Supervision

The general agreement that supervisors should have training, or at least access to supervisor training courses, is in line with modern trends 17 in recognition of the many new demands that are now made of the supervision process. Studies that show the benefit or otherwise of such courses is at present lacking, although a longitudinal analysis of the KI Exit Poll associates positive trends with the implementation of supervisor training.

### The thesis

In UK, the traditional 250–300 page thesis provides the PhD student with the opportunity for detailed presentation of the experiments performed and the methodology that has been used, together with extensive review of the literature and discussion of the results. The extent of detail can far exceed what is possible in any papers that may arise from the work, and allows for discussion of failed experiments and dead‐end research experimentation. Published papers can thus be seen as a distraction from the main purpose of the PhD training 18. In Scandinavia, with its emphasis on the papers resulting from the PhD project, there has historically been a tendency for reduced emphasis on the actual thesis, which nonetheless contains a review of the literature, critique of the methodology and discussion of results. In practice, as indicated above, many Scandinavian theses are rather short, and the defence tends to concentrate primarily on the papers. Thus, in UK, the emphasis is on research training *per se*, while in Scandinavia there is more emphasis that the research training will lead to publishable results. It may be added that at the Karolinska, the aim in the future is to focus on the training of the PhD student with the knowledge that only a fraction of the student's efforts will actually be represented in the figures included in the published papers/manuscripts.

An important factor that supports the Scandinavian emphasis on publication is that the publication output of institutions is a direct parameter in the central allocation of research funds. Supervisors and PhD students also place emphasis on publications to provide them, respectively, with support for funding applications and with a good CV and good employment prospects. Data about the extent to which PhD students contribute to the research output of institutions is sparse, but at the Karolinska a recent investigation concluded that over 50% of the research publications included a PhD student and that the average impact factor was slightly higher than that of papers not including a PhD student. A detailed study of the number of papers published in 2000–2007 in Quebec, Canada, showed that one‐third of the papers were based on PhD projects 19. Published papers are of course also of great importance in the UK and an indirect source of income, but PhD students are also a source of income and large emphasis is thus placed on excellence of PhD training. The work done during PhD projects will form part of the supervisor's research output, but not necessarily as independent articles.

The difference in approach concerning the thesis has significant practical consequences. UK students who apply for postdoc positions in Europe may be disadvantaged if they do not have publications; students who have experience in publishing their work are preferred. Scandinavian students who apply for positions in UK may be disadvantaged, since the contribution of a student to a multiauthored publication is not clear, and it is also not clear whether a student has the technical ability to do laboratory work. Conversely, in the UK and other countries following the UK tradition, the thesis is in itself a major qualification for a postdoc position, while in Scandinavia it is the excellence of the publications which makes the PhD graduate competitive. It may therefore be that both UK and Scandinavia are rejecting good PhD graduates due to lack of knowledge of the basis for their respective qualifications.

### The assessment process

In the UK, in keeping with the original Humboldt concept, the primary outcome of PhD training is the thesis – it is the thesis that is examined and it is the thesis that the PhD student has to defend. This is a highly technical process and one that is most efficiently performed with the traditional UK method of having two independent examiners discussing alone with the PhD student. As described above, the Scandinavian approach is traditionally based on assessing the papers that have been produced during the PhD training, with emphasis on the ability of the PhD student to be able to defend his or her work in open debate. Multiauthorship, which is the current norm in scientific publishing, is not seen as a problem providing the precise role of the PhD student has been explained.

To the extent that there appear to be different goals of PhD training, the two approaches to assessment are appropriate. The closed nature of the UK examination is integral to this being an academic evaluation of a document written by the PhD student, while the open nature of the Scandinavian examination is integral to the PhD student being able to contribute to the scientific environment.

Despite the different backgrounds, there are arguments for convergence. For example, in the UK a public lecture could be introduced as part of the examination process, and more emphasis could be placed on publication. In Scandinavia, a closed detailed examination of the thesis could be introduced in addition to the traditional public defence. Furthermore, consideration could be given to switching emphasis from publication‐at‐all‐costs to deeper research projects. There is also an apparent need both in UK and in Scandinavia for more transparency in the criteria used by assessment committees. The use of formative assessment practices, including annual feedback with regard to progress in achievement of intended learning outcomes, is common practice at KI.

### Financing

The different financial models may be expected to affect the expectations that PhD student and supervisor have from PhD training. The package offered by UK PhD programmes is clear‐cut: 3 years' work in the laboratory learning research techniques and writing this up in a thesis, independent of whether the work is publishable. The UK PhD has a long tradition and has a strong basis 20, and PhD students are prepared to pay for the package. For UK and EU PhD students, the total cost of doing a PhD (including living expenses) is perhaps relatively modest and covered by their stipends, but for other PhD students, the total cost may be £100 000 or more. The large number of applications for PhD positions attests to this price being acceptable.

In contrast, the Scandinavian package is to some extent less clear‐cut since it is primarily dependent on doing publishable work, even though publication cannot be guaranteed. Nevertheless, at Bergen and Karolinska, there is also considerable competition for the available PhD fellowships, although it should here also be taken into account that the positions have the added attraction that PhD training is provided free and PhD students receive a salary almost corresponding to junior faculty.

### Employment

Several studies, both in the UK and in Scandinavia, have shown that only a minority of PhD students find final employment in a permanent academic position, and indeed, only a minority proceed to regular research positions 9, 14. This naturally raises questions as to whether the PhD is a useful form of training for the student concerned. However, recent studies have indicated that PhD graduates have job satisfaction 21, and higher salaries and higher levels of employment than the general population and indeed higher than for graduates with only master's degrees.

Career advice is a growing responsibility for graduate schools. Given the high employment rate of PhD graduates, this is not perhaps needed but could be a way of ensuring that PhD graduates are aware of, and make best use of, the competences they have developed.

### Comparison with United States

In the United States 7, admission to a PhD programme requires a bachelor examination, but the first 2 years is spent primarily on academic courses leading up to a qualifying examination, not dissimilar to a master's. Only after passing the qualifying examination does the student start the thesis project. Thus, although the admission requirements are similar to those in the UK, the overall requirements are more similar to those in Scandinavian. A procedure that differs from both the UK and Scandinavia is the use of a Thesis Committee. This committee chosen largely by the student plays a major role in monitoring the project with formal meetings about twice a year, giving advice to the student (and the supervisor) about how the project should develop. Importantly, it is the Thesis Committee that decides at the end of the project whether the PhD thesis should be approved. The PhD student will normally have published at least one article, and often more (similar to Scandinavia), but the length of the thesis can be over 200 pages and it is the thesis that is the basis for granting a degree (similar to UK).

## Conclusions

Graduate studies in the UK have a solid base as an academic research training programme for those who have completed their bachelor studies. The primary aim is to produce a thesis which is then evaluated as an academic document. UK graduate studies are popular with a large number of applications from overseas despite the considerable financial expenses involved. In these countries, the thesis is in itself a valued qualification. Graduate studies in Scandinavia are for those who will normally already have completed a substantial research project during their master's studies, and the main aim is to be responsible for projects where they may have technical and other assistance, and which will be published. Public support for graduate studies in Scandinavia is predicated on the expectation that doctoral graduates will become drivers for development of the ‘knowledge society'. These differences in approaches have resulted in the substantial differences between UK and Scandinavian graduate programmes indicated in this paper. Nevertheless, the overarching aim of both systems is to equip a new generation of professionals with the skill sets required to take their place in society through training in research. Reflection of the ways that different programmes aim to achieve this common goal might lead to improvement and illustrates that graduate programmes have much to learn from each other's experiences. It is of particular of concern that UK institutions may not appreciate the value of Scandinavian PhD theses (often based on multiauthored publications) and that Scandinavian institutions may not appreciate the value of UK theses (often without accompanying publications). It may be hoped that analyses such as those found in this paper may aid understanding of the basis for UK and Scandinavian theses, and the value that can be placed on each.

## Conflict of interest

The authors declare no conflict of interest.

## Author contributions

MJM conceived and designed the project and wrote the first draft of the paper. Other authors obtained the data and took part in revision and approval of the final draft.

## Supporting information


**Table S1**. Comparison of responses to ORPHEUS Self‐evaluation questionnaire (5) of four PhD programmes at (1) School of Medicine, Cardiff University; (2) Institute of Integrative Biology, University of Liverpool; (3) Faculty of Medicine and Dentistry, University of Bergen; (4) Karolinska Institutet, Stockholm.

**Table S2**. Responses to ORPHEUS Self‐evaluation questionnaire.
