# Supplementary material for: A comparison of doctoral training in biomedicine and medicine for some UK and Scandinavian graduate programmes: learning from each other
Source: FEBS Open Bio. 2019 Mar 30;9(5):830–9. doi: 10.1002/2211-5463.12629 (PMC6487698; doi:10.1002/2211-5463.12629)
Supplement: Supplementary file 1 — Table S1. Comparison of responses to ORPHEUS Self‐evaluation questionnaire (5) of four PhD programmes at (1) School of Medicine, Cardiff University; (2) Institute of Integrative Biology, University of Liverpool; (3) Faculty of Medicine and Dentistry, University of Bergen; (4) Karolinska Institutet, Stockholm. [file FEB4-9-830-s001.docx]

Table S1. Comparison of responses to ORPHEUS Self-evaluation questionnaire (1) of four PhD programmes at (1) School of Medicine, Cardiff University; (2) Institute of Integrative Biology, University of Liverpool; (3) Faculty of Medicine and Dentistry, University of Bergen; (4) Karolinska Institutet, Stockholm.

The questionnaire is based on the recommendations of the ORPHEUS 2016 edition of Best Practices document (1) and the full responses are shown in Table S2.

Explanation of Table.

Column 1 shows line number in Table S2 and the ORPHEUS Best Practices reference (BR, basic recommendation; QD, quality development).

Column 2 shows an abbreviated form of the recommendation.

Column 3 summarizes the responses of the four programmes.

Column 4 compares responses where ‘0’ indicates strong similarity, ‘1’ indicates small differences, and ‘2’ indicates substantial differences.

| Line #  and Ref. | Brief description of recommendation | Responses of the four programmes | Comparisons |
| --- | --- | --- | --- |
| **1. Research environment** | |  |  |
| #1: BR 1.1 | There should be a strong research environment around every PhD project, either within the institution or within collaborating institutions. | All programmes have strong research environments. | 0 |
| #2: BR 1.2 | Facilities should be compatible with the requirements of completing the PhD project. | All programmes have facilities for completing PhD projects. | 0 |
| #3: BR 1.3 | Research should be consistent with international ethical standards. | All programmes comply with international ethical standards. | 0 |
| #4: BR 1.4 | There should be provision for allowing PhD candidates to perform part of their programme in another institution, national and abroad. | All programmes have this possibility, but relatively few candidates take advantage of it. | 0 |
| #5: QD 1.1 | Institutions lacking facilities/expertise could collaborate with stronger institutions to obtain these. | All programmes collaborate with other programmes. | 0 |
| #6: QD 1.2 | Possibilities for joint and double degrees could be explored. | - Cardiff: work in Progress. - Liverpool: one programme with Thai programme, 3 candidates have followed this. - Bergen: regulations allow this. - Karolinska: these types of degrees are established. | 2 |
| **2. Outcomes** | |  |  |
| #7: BR 2.1 | PhD programmes should provide PhD candidates with competences to become qualified and independent researchers, according to principles of good research practice. | - Cardiff: Research Plan and Development Needs Analysis. - Liverpool: Vitae Researcher Development Framework. - Bergen: consistent with Salzburg. - Karolinska: developing as an independent researcher is a basic Intended Learning Outcome (ILO). All programmes have provide re-evaluation after one or 1½ years.   UK approach is more formalized, while in Scandinavia it is left more to the candidate and the supervisor to decide what is needed.  Scandinavian programmes provide a wide range of courses, and candidates will typically follow 6 months of coursework. Coursework appears to be less in UK. | 1 |
| #8: BR 2.2 | A PhD degree should also be of benefit in a career outside academic or clinical research (problem solving, analysis, evaluation, technology transfer etc.). | All programmes believe that their programmes provide candidates for employment outside of academia, but none has formal mechanisms for providing all candidates with career advice. | 0 |
| #9: BR 2.3 | The outcomes for PhD candidates with a background in medicine or other professional training are the same as for any other PhD. | All programmes expect the same standard of all candidates regardless of background. | 0 |
| **3. Admission policy and criteria** | |  |  |
| #10: BR 3.1 | PhD candidates should be selected on the basis of a competitive and transparent process. | - Cardiff: all projects are advertised and applicants selected following interview and interactions with department. - Liverpool: In general as for Cardiff, but self-financed candidates are interviewed to assess ability rather than competition for funding. - Bergen: applications for UiB stipends are dealt with on a competitive basis; applications from students with alternative funding may be accepted following ‘comprehensive evaluation of the project description, the applicant's formal qualifications, sufficient resources for the realisation of the research project and the plan submitted for the research training’. - Karolinska: all projects are advertised and applicants selected by a Panel at department level.   In all cases, where the programmes or other funding bodies are offering stipends, the application and selection process is transparent and competitive. Where there is alternative funding applications may be accepted without (direct) competition following evaluation of the candidate’s ability and the quality of the project. | 1 |
| #11: BR 3.2 | Applicants for PhD programmes should have an educational level corresponding to a master’s degree. | - Cardiff, Liverpool: applicants should have a bachelor’s degree (First or Upper Second) or Lower Second and master’s degree. - Bergen, Karolinska: applicants should have a 5-year master’s degree (this includes medical and other professional degrees). | 2 |
| #12: BR 3.3 | Before enrolment or at clearly defined times during the programme, the institution should evaluate and approve:  - Scientific quality of the project,  - Likelihood to complete within normal timeframe,  - The possibility for candidate to provide creative input,  - Qualifications of supervisors. | All programmes require approval of the project by an independent panel prior to admission. | 0 |
| #13: BR 3.4 | A PhD programme should only be initiated when the resources for completion are available. | All programmes require that financing of the PhD programme is in place before admission. | 0 |
| #14: QD 3.1 | In choosing PhD candidates, the applicants’ potential for research ought to be considered, not just past academic performance. | All programmes may take account of previous research experience although such experience is not an absolute requirement. In UK previous research experience will be a competitive advantage for admission. In Scandinavia such experience will normally be part of their master’s degree. | 0 |
| #15: QD 3.2 | Projects ought to be externally assessed by written project description or presentation to panel of independent scientists. | All programmes require independent assessment of projects, although this may be at department level (Bergen, Karolinska). | 0 |
| #16: QD 3.3 | If the PhD candidate is obliged to obtain extra income, it ought to be ensured that the PhD candidate has the necessary time to complete the programme. | All programmes allow extra time if the candidate has other employment while doing their PhD. | 0 |
| **4. PhD training programme** | |  |  |
| #17: BR 4.1 | Programmes should be based on original research, courses and other activities which include analytical and critical thinking. | All programmes are based on original research. It is expected that this together with e.g. journal clubs, participation in national and international meetings, manuscript writing will provide training in analytical and critical thinking. | 0 |
| #18: BR 4.2 | Programmes should be performed under structured supervision. | All programmes have structured supervision | 0 |
| #19: BR 4.3 | Programmes should ensure that PhD candidates have appropriate training in ethics and responsible conduct of research. | All programmes require that candidates follow a course in ethics. | 0 |
| #20: BR 4.4 | Programmes should have clear 3-4 year timeframe. Extensions should be possible but limited and exceptional. | Nominal time frames are 3-4 year (UK), 3-4-year (Bergen), 4-year (Karolinska). Extensions are possible and normal (e.g. average times Cardiff: 3.6 year; Liverpool: 3.9 years; Bergen: 4.7 years; Karolinska: 4.5 years). | 0 |
| #21: BR 4.5 | Programmes should include relevant activities not directly related to the project (e.g. courses, journal clubs, participation in conferences, seminars and workshops, including preparation time) totalling about 15% of the whole programme. A substantial part should be concerned with training in transferable skills. (NB. ‘training’ can be liberally interpreted as all scientific activities not directly related to the project, e.g. journal clubs, conferences, etc.). | - Cardiff, Liverpool: training courses are available but are not an integrated part of a PhD programme. - Bergen, Karolinska: training courses etc. corresponding to 30 ECTs (6 months) are required for all candidates. | 2 |
| #22: BR 4.6 | PhD programmes that are performed in parallel with clinical or other professional training should have the same time for research and course work as any other PhD. | This is the case for all programmes. | 2 |
| #23: BR 4.7 | Progress of PhD candidates should be continuously assessed by the institution throughout the PhD. | All programmes have at least annual assessments. | 0 |
| #24: QD 4.1 | For PhDs performed by clinicians, leave-of-absence from clinical duties could be provided for the PhD part of such programmes | All programmes allow this. | 0 |
| #25: QD 4.2 | PhD programmes could where relevant have an element of interdisciplinarity. | Programmes encourage this. Info from Karolinska lacking. | 0 |
| **5. Supervision** | |  |  |
| #26: BR 5.1 | Each PhD candidate should have a principal supervisor and normally at least one co-supervisor. | For all programmes, one principal supervisor and one or more co-supervisors. | 0 |
| #27: BR 5.2 | The number of PhD candidates per supervisor should be compatible with the supervisor’s workload. | - Cardiff: a supervisor may have up to six candidates. - Liverpool: a “limited” number. - Bergen: up to 14 candidates. - Karolinska: no formal limit.   Three is the norm for all programmes. | 0 |
| #28: BR 5.3 | Supervisors should be scientifically qualified and active scholars in the field concerned. | For all programmes, supervisors must have a doctoral degree or equivalent academic competence within the subject area, and be an active researcher. | 0 |
| #29: BR 5.4 | Supervisors should have regular consultations with their PhD candidates. | - Cardiff: ‘in accordance with an agreed frequency’. - Liverpool: ‘supervisory meetings annually full-time/part-time. In practice most supervisors have ‘open door’ policy and regular group meetings so meet much more frequently’. - Bergen: ‘expected to meet several times per month’. - Karolinska: ‘daily, weekly or monthly physical meetings’. | 0 |
| #30: BR 5.5 | It should be ensured that training for all supervisors and potential supervisors is available. | - Cardiff: 226 academics have received supervisor training over the past two years. - Liverpool: supervisor training required for new staff. Course are under re-development. - Bergen: compulsory e-learning course for all principle supervisors and an annual 1-day seminar for all supervisors. In addition, a supervisor training course is available and there are regular 2-hour supervision seminars. - Karolinska: 1-week ‘basic’ training course compulsory for all new supervisors. Other courses available. | 2 |
| #31: BR 5.6 | The supervisor-candidate relationship is the key to a successful PhD programme. There should be mutual respect and shared responsibility. | - Liverpool, Bergen and Karolinska: supervisors and candidates are matched by mutual consent. - Cardiff: matchmaking often based on the student having an interest in a particular project.   All programmes recognize that conflicts sometimes arise, and have procedures for helping to resolve conflicts. | 0 |
| #32: BR 5.7 | Institutional assistance should be provided for career development. This should be continuous, starting from the time of enrolment. | - Cardiff: there is career assistance at induction and this continues throughout the programme. - Liverpool: assistance with career assistance is available. - Bergen: candidates are encouraged to make career plans at induction, courses are available, and there is an annual Career Day. - Karolinska: Central career service that organises professional development courses available to all early stage researchers, career days and subject-specific seminars. It is also the remit of Karolinska supervisors to discuss career planning with their PhD students, and the first part of the annual assessment form specifically addresses this aspect.   For all programmes, the supervisor has a responsibility to provide career assistance. | 1 |
| #33: QD 5.1 | Responsibilities of each supervisor ought to be explicit. | This is the case for all programmes. | 0 |
| #34: QD 5.2 | Supervisors ought to have broad local and international scientific networks. | For all programmes, the required solid publication record is taken to imply that supervisors have scientific networks. | 0 |
| #35: QD 5.3 | Supervisors ought to assist with career development. | Yes. See BR 5.7. | 0 |
| #36: QD 5.4 | Institutions could consider having contracts on the supervision process, signed by supervisor, PhD candidate and head of graduate school. | All programmes have this. | 0 |
| #37: QD 5.5 | The principal supervisor, at least, ought to have some formal training as supervisor. | See BR 5.5. Cardiff and particularly Karolinska have capacity for training large numbers of supervisors. At Karolinska, it is a requirement for new supervisors that they have had supervisor training. Supervisor training courses at Bergen appear more limited. Liverpool: supervisor training required for new staff. Course are under re-development.. | 2 |
| #38: QD 5.6 | Supervisors could where possible also act as co-supervisors for PhD candidates at other graduate schools. | This is possible for all programmes. | 0 |
| #39: QD 5.7 | Graduate schools ought to consider appointing a mentor or equivalent for each PhD candidate, in addition to the supervisor team, to discuss programmes from another aspect than the science topic alone. | - Cardiff: mentors are appointed in year 1. - Liverpool: all candidates have assessors who can act as mentors. - Bergen: does not have such an arrangement. - Karolinska: all students have a mentor. | 2 |
| **6. PhD thesis** | |  |  |
| #40: BR 6.1 | The PhD thesis should be the basis for evaluating if the PhD candidate has acquired independent research skills and can evaluate work done by others. | For all programmes, the thesis and the examination/defence are the basis for making these assessments. | 0 |
| #41: BR 6.2 | The benchmark for a PhD thesis in health sciences is the equivalent of three in extenso papers in scientific peer-reviewed international journals. Manuscripts are also acceptable. It is the task of the assessment committee to determine if the material demonstrates 3-4 years of research at international level. | - Cardiff, Liverpool: the thesis does not have to include papers and it is the thesis monograph that is evaluated. - Bergen, Karolinska: the thesis will normally contain 2-3 papers (published or manuscripts) in addition to the overview.   For all programmes it is the Assessment Committee that is responsible for determining if the work done corresponds to 3-4 years of research at international level. | 2 |
| #42: BR 6.3 | In defining the benchmark for a PhD thesis, the assessment committee should take account of the provisos listed in the Annotations, for example the annotation indicating that fewer than three papers may be accepted if published in highly rated journals. | - Cardiff, Liverpool: This recommendation is not strictly relevant. - Bergen: The number of articles will depend upon the extent and quality of each article, and on the candidate’s contribution. If the candidate has put an unusually large amount of work into one article, and that article is of a very high standard, the number of articles may be reduced. It is the task of the evaluation committee to determine if the material demonstrates 3 years fulltime of research at international level. - Karolinska: The pre-assessment by the examination committee before the formal thesis defence is approved centres on conforming that the work presented is what should be expected for a 4-year time equivalent period of research training. The number and quality of the component research papers/manuscripts are thus considered at this time, and provision for fewer, higher impact publications can therefore be accepted at this stage. | 2 |
| #43: BR 6.4 | In addition to papers, the thesis should include a full literature review and full account of aims, method, results, discussion and conclusion. | - Cardiff, Liverpool: this is included in the bound thesis. - Bergen, Karolinska: such an overview is provided in addition to papers. | 0 |
| #44: BR 6.5 | If the thesis is presented in other formats (e.g. as single monograph), the assessment committee should ensure equivalence to the above benchmark. | - Cardiff, Liverpool: monographs are the norm. - Bergen, Karolinska: monograph format is allowed but rare. | (0) |
| #45: BR 6.6 | A PhD thesis in clinical medicine should meet the same standards as other PhD theses . | Yes, for all programmes. | 0 |
| #46: QD 6.1 | The thesis ought to be written and optimally also defended in English, unless national regulations stipulate otherwise. An abstract of the thesis should be published in English. | For all programmes, English is the norm for both the thesis and the examination/defence. Cardiff: Welsh is allowed. Bergen, Karolinska: Scandinavian languages are allowed. | 0 |
| #47: QD 6.2 | If articles/manuscripts are joint publications, co-author statements ought to document that the PhD candidate has made a substantial and independent contribution. Ownership of results from PhD studies ought to be clearly stated. | This is the case for all programmes. | 0 |
| #48: QD 6.3 | PhD theses ought to be published on the graduate school’s homepage, preferably in extenso. If patent or copyright legislation prevent this, at least abstracts of the theses ought to be publicly accessible. | This is the case for all programmes. | 0 |
| #49: QD 6.4 | There could be a lay summary of the thesis in the local language. | - Cardiff, Liverpool: lay summaries are not made. - Bergen: lay summaries in form of press release for all theses. - Karolinska: common but not a requirement. | 2 |
| **7. Thesis assessment** | |  |  |
| #50: BR 7.1 | Acceptance of a PhD thesis should include acceptance of both written thesis and a subsequent oral defence. | This is the case for all programmes. | 0 |
| #51: BR 7.2 | PhD degrees should be awarded by the institution on the recommendation of the assessment committee which has evaluated the thesis and the oral defence. | This is the case for all programmes. | 0 |
| #52: BR 7.3 | The assessment committee should consist of established and active scientists without connection to the milieu where the PhD was performed and without conflict of interest. Min. two should be from another institution. | This is the case for all programmes. There are differences in the format of the assessment.   - Cardiff, Liverpool: the thesis is assessed in closed session viva voce by two independent examiners. - Bergen: the candidate is assessed by two independent opponents, who assess first the thesis and subsequently the public defence. - Karolinska: the thesis and the public defence are assessed by an Examination Board of at least three experts, one of whom is from another (Swedish) programme. The defence consists of a discussion between the candidate and an invited ‘faculty opponent’ who may be from another country. | (0) |
| #53: BR 7.4 | The supervisor should not be a member of the assessment committee. If local regulations require this, the supervisor should not have a vote. | This is the case for all programmes. | 0 |
| #54: BR 7.5 | If the assessment of the thesis/defence is negative, the PhD candidate should normally be given an opportunity to rewrite/an additional defence. | This is the case for all programmes. | 0 |
| #55: BR 7.6 | The oral examination should be detailed enough to ensure that the thesis is the candidate’s own work, that the intended training goals have been achieved, and that the candidate is able to put the results into scientific context. | - Cardiff, Liverpool: the viva voce is an oral defence/examination of the thesis NOT a formality. The PhD candidate does not give a formal lecture. - Bergen: The candidate gives both a trial lecture on a topic related to the thesis (to demonstrate good knowledge about related research), and then a lecture at the defence, followed by 2-3 hours of questioning by the opponents. Opposition ex auditorio is possible. - Karolinska: The formal thesis defence includes a research seminar summarising the work conducted by the candidate. Thereafter the faculty opponent and subsequently the 3 examination committee members ask questions to assess the candidate’s attainment of learning outcomes. It is inherent from this question-answer-discussion session whether the candidate has been sufficiently trained and has conducted the research him- or herself. | 2 |
| #56: QD 7.1 | The oral defence ought to be open to the public. | - Cardiff: not open to the public, only the examiners are present. - Liverpool: not open to the public; only the candidate and the 2 examiners can attend (plus an independent chair if, for example, the thesis is a re-submission) - Bergen, Karolinska: open to public. | 2 |
| #57: QD 7.2 | Where possible at least one member of the assessment committee could be from another country. | - Cardiff, Liverpool: ‘few’. - Bergen: one member should be international. - Karolinska: often an international scientist acts as the ‘faculty opponent’. | 2 |
| #58: QD 7.3 | Apart from the thesis, the institution ought to ensure that sufficient transferable skills have been acquired during the PhD programme. | - Cardiff, Liverpool: it is expected that candidates have acquired transferable skills, but there is no formal check on this. - Bergen, Karolinska: candidates must have satisfactorily completed training courses in transferable skills. | 2 |
| #59: QD 7.4 | The competences developed during the PhD programme could be documented in a portfolio. This documentation could be evaluated by the assessment committee and form part of their decision concerning the award of the PhD degree. | - Cardiff, Liverpool: facility available for candidates to record their portfolio. - Bergen: diploma supplement to thesis listing courses taken. - Karolinska: a personal portfolio will soon supplement the current official transcript record.   There is no formal assessment of the portfolio at any programme. | (0) |
| **8. Structure of Graduate School** | |  |  |
| #60: BR 8.1 | The graduate school should have sufficient resources for proper conduct of PhD programmes. This includes resources to: Support admission of PhD candidates, implement the PhD programmes of the PhD candidates enrolled, assess PhD theses and award PhD degrees. | This is the case for all programmes. At Liverpool, the administration for doctoral training is being consolidated with transfer of staff from departments to a central ‘Liverpool Doctoral College, LDC’. | 0 |
| #61: BR 8.2 | The graduate school should have a website in English and possibly also the national language including transparent information about the content of PhD programmes and the policies of the graduate school. | This is the case for all programmes. | 0 |
| #62: BR 8.3 | Credit should be given for courses taken elsewhere or other relevant experience. | This is the case for all programmes. For Liverpool, such courses must be taken during the PhD programme; for other programmes, courses taken previously may be considered for credit. | 0 |
| #63: QD 8.1 | There ought to be procedures for regular review and updating of the structure, function and quality of PhD programmes, including both supervisor and candidate feedback. | This is the case for all programmes. | 0 |
| #64: QD 8.2 | Representatives of the PhD candidates ought to interact with the leadership of the graduate school regarding the running of the graduate school. Candidate organisations ought to be encouraged and facilitated. | - Cardiff: There is a ‘Vice President Postgraduate Students’ who represents the 12,500 postgraduates in development of programmes. - Liverpool: there are candidate representatives in the Faculty PGR committee. - Bergen: there are candidate representatives in the faculty programme board. - Karolinska: Candidate representatives in all relevant committees. | (0) |
| #65: QD 8.3 | PhD candidates ought to have rights and duties commensurate with the value (to the institution) of the research performed. | Scandinavian programmes require their PhD students to be paid salaries (either programme/government stipends or support from other sources) at a level corresponding to junior faculty. The question is not relevant for UK, since PhD programmes are considered as training. | 2 |
| #66: QD 8.4 | There ought to be an appeal mechanism allowing PhD candidates to dispute decisions concerning their programmes and thesis assessment. | This is the case for all programmes. | 0 |
| #67: QD 8.5 | Confidential candidate counselling concerning e.g. the PhD programme, supervision, as well as personal matters ought to be offered by the graduate school. | - Cardiff, Liverpool: confidential counselling is available. - Bergen: ‘PhD co-ordinators’ in each department can provide advice. - Karolinska: candidates have ‘external mentors’ and may also consult with university doctoral ombudsman for confidential advice. | 2 |
| #68: QD 8.6 | Graduate schools could consider having a thesis committee for each PhD candidate that monitors the progress of the PhD candidate through meetings with the PhD candidate and the supervisors. | - Cardiff, Liverpool: there are independent panels that assess candidate progress. - Bergen, Karolinska: there is a half-time independent review committee. | 1 |

References

1. ORPHEUS. ORPHEUS self-evaluation questionnaire2017. Available from: <http://www.orpheus-med.org/>.
