# Supplementary material for: A comparison of doctoral training in biomedicine and medicine for some UK and Scandinavian graduate programmes: learning from each other
Source: FEBS Open Bio. 2019 Mar 30;9(5):830–9. doi: 10.1002/2211-5463.12629 (PMC6487698; doi:10.1002/2211-5463.12629)
Supplement: Supplementary file 2 — Table S2. Responses to ORPHEUS Self‐evaluation questionnaire. [file FEB4-9-830-s002.docx]

**Table S2. Responses to ORPHEUS Self-evaluation questionnaire**

Table shows responses to ORPHEUS Self-evaluation questionnaire from (1) School of Medicine, Cardiff University; (2) Institute of Integrative Biology, University of Liverpool; (3) Faculty of Medicine, University of Bergen; (4) Karolinska Institutet, Stockholm. The Self-evaluation questionnaire (<http://www.orpheus-med.org/images/stories/documents/ORPHEUSSelf-evaluationform2016.pdf>) is based on the ORPHEUS Best Practices document 2016: (<http://www.orpheus-med.org/images/stories/documents/ORPHEUS%20Best%20Practices%20DOCUMENT%202016.pdf>)

Column on right shows if the four programmes have generally similar practices (0), minor differences (1), significant differences (2).

| Line #  and Ref. | Brief description (with ORPHEUS reference to 2012 edition of Best Practices document) | Cardiff | Liverpool | Bergen | Karolinska | 0. no diff.  1.small diff.  2. sign. diff. |  |  |  |  |  |  |
| --- | --- | --- | --- | --- | --- | --- | --- | --- | --- | --- | --- | --- |
|  | | ***1. Research environment** | | | | | |  |  |  |  |  |
| #1  BR  1.1 | There should be a strong research environment around every PhD project, either within the institution or within collaborating institutions. | Research Excellence is a central theme of the School’s vision and ambition. Each of our Research Divisions have thematic areas of strength which are supported by infrastructure and professional support services.  Division of Cancer & Genetics: Research for the benefit of patients affected by cancer and by inherited disease in Wales and beyond, and at all points in the patient pathway. Research themes: Genetic and genomic medicine, haematology and solid cancers. Key paper: 10-Year Outcomes after monitoring, surgery, or radiotherapy for localized prostate cancer. New England Journal of Medicine  Division of Infection & Immunity: Research that extends from basic mechanisms of infection and immunology to translation directly impacting diagnosis, management and prevention of disease. Research themes: Infection, inflammation & Immunology. Key paper: Long-term outcomes of imatinib treatment for chronic myeloid leukaemia. New England Journal of Medicine  Division of Population Medicine: Research to improve the health of individuals through research, innovation, and teaching focused on patient populations and the health systems affecting their care. Key paper: Effectiveness of a nurse-led intensive home-visitation programme for first-time teenage mothers (Building Blocks): a pragmatic randomised controlled trial. The Lancet  Division of Psychological Medicine and Clinical Neurosciences: Research to understand the fundamental mechanisms underlying major psychiatric and neurological disorders. Research Themes: Psychosis and major affective disorders, developmental disorders and neurodegenerative disorders. Key Paper: Lithium use in pregnancy and the risk of cardiac malformations. New England Journal of Medicine.  Clinical Trials Research key paper: Assessment of minimal residual disease in standard-risk AML. New England Journal of Medicine.  The University participates in the biennial Postgraduate Research Experience Survey (PRES), co-ordinated by the Higher Education Academy (HEA) and uses the outcomes to inform institutional, College and School action planning. The PRES provides a key source of information for the Annual Review and Enhancement quality process. | The University of Liverpool is a member of the Russell group of research-intensive University (see <https://www.liverpool.ac.uk/research/our-research/> and <https://www.liverpool.ac.uk/researcher-hub/positive-research-environment/> ). In the 2014 Research Excellence Framework assessment of all UK Universities undertaken for the UK government, 81% of the University of Liverpool’s research was ranked world-leading and internationally excellent. The University’s three Faculties (Humanities and Social Sciences; Science and Engineering; Health and Life Sciences) undertake research across a wide range of subject.  There are approx. 2000 PhD students. The University re-organised its graduate school in 2015, introducing Liverpool Doctoral College (<https://www.liverpool.ac.uk/study/postgraduate-research/liverpool-doctoral-college/> ).This has made changes in approach to aspects of training for candidates as well as career planning (mentioned when appropriate below).  This document will focus on the Institute of Integrative Biology (IIB), one of the five research-orientated Institutes in Health and Life Sciences with about 150 PhD students. It has 4 research themes: Dynamics and management of host-microbe interactions; Molecular basis of therapeutic targeting; From genomes to biological systems; Adaptation to environmental change. See [www.liverpool.ac.uk/integrative-biology/research](http://www.liverpool.ac.uk/integrative-biology/research) including links to publications | For a description of research areas covered by the Faculty, see [attachment 2](file:///C:\Users\au184\AppData\Local\Microsoft\Windows\INetCache\Content.Outlook\0EYU0S41\Attachment%202%20-%20Research%20areas%20(BS1.1).docx)**. ***  For a list of key papers, see [attachment 3](file:///C:\Users\au184\AppData\Local\Microsoft\Windows\INetCache\Content.Outlook\0EYU0S41\Attachment%203%20-%20Key%20papers%20(BS1.1).docx). * The papers were selected by the relevant departments and have all been included in a PhD thesis in the last few years.  * Will be made available later | There are 22 Departments within KI encompassing the following research areas: Neuroscience, Infection Biology, Inflammation, Cell and Molecular Biology, Physiology & Pharmacology, Clinical Medicine (all specialisations e.g. surgery, dentistry), , Public Health, Healthcare sciences, Biosciences, Laboratory Medicine, Epidemiology & Biostatistics, , Developmental biology, Environmental Medicine, Medical Pedagogics.  See [www.ki.se](http://www.ki.se/) (pull-­‐down menu to the right-­‐hand-­‐side) for more information about specific Departments.  See [http://ki.se/ki/jsp/polopoly.jsp?l=en&d=135](http://ki.se/ki/jsp/polopoly.jsp?l=en&amp;d=135) for more information about research at KI.  Quality of the whole research environment for the doctoral candidate is assessed by the Departmental Admissions Board at the time of application for PhD studies. | 0 |  |  |  |  |  |  |
| #2  BR  1.2 | Facilities should be compatible with the requirements of completing the PhD project. | Each Division has access to high-tech equipment capable of generating high quality, competitive, research outputs.  Open Access IT rooms across the campus, some of which are open 24 hours a day, 7 days a week, are freely available.  Modern libraries offer print and online resources providing flexible study and research environments, with long opening hours, including evenings and weekends throughout the year  Advanced Research Computing Advanced Research Computing (ARCCA) provides, co-ordinates, supports and develops advanced research computing services for our researchers and research students.  In addition to the facilities provided by individual divisions, post-graduate research students are offered centrally-located social and study facilities. Post-graduate study zones for the exclusive use of post-graduates in Cathays and Heath Park Campus’ and are accessible 24/7. | Facilities in IIB include centres for genomics, proteomics, cell imaging, synthetic biology, NMR, X-ray structure and animal behaviour (see <https://www.liverpool.ac.uk/integrative-biology/facilities-and-services/>). The Institute has 8 multi-user laboratories and office space used by the research groups of the approx. 75 PIs, plus a separate NMR centre and animal behaviour centre. Each PhD student is provided with a desk, computer and lab space.  The University library system ([www.liverpool.ac.uk/library](http://www.liverpool.ac.uk/library)) gives electronic and physical access to the scientific literature.  Other facilities at the University come from collaboration with other Institutes (E.g. Institute of Translational Medicine; Institute of Infection and Global Health) in the same Faculty, from Departments (e.g. Chemistry, Mathematics, Environmental Sciences) in Faculty of Science and Engineering and outside the University (e.g. Daresbury Laboratory; Diamond; other universities (worldwide); industry; research centres) | Facilities available for PhD candidates are divided into three levels:   1. [**The Division of Research Management**](http://www.uib.no/en/fa), which provides service and support to the UiB’s central management, the Faculties, departments, research groups and individuals. It is also The Division of Research Management who has the overall responsibility for the PhD programme at the UiB. 2. [**The Faculty**](http://www.uib.no/en/mofa/64913/doctoral-education-faculty-medicine-and-dentistry), which has the overall responsibility for administering the PhD programme at the Faculty, and provides service and support to the departments, research groups and individuals affiliated with the Faculty.   [**The Programme Board**](http://www.uib.no/en/mofa/93296/programme-board-phd-programme-pfu-faculty-medicine-and-dentistry) is organised under the Faculty, and among its main purposes are to ensure scientific coordination and high quality in the PhD programme at the Faculty of Medicine.   1. [**The Departments**](http://www.uib.no/en/about/79366/organisation-chart-university-bergen), which are responsible for following up and provide academic and administrative support to their candidates, and to ensure that their candidates have the necessary facilities available to successfully complete the PhD project within the set timeframe. | Whenever a PhD student applies for admission the working environment, including the psychosocial environment, and the physical space and facilities therein or available to the project, are judged by the admissions committee. This not only encompasses lab spaces with relevant technical apparatus but also office facilities, including computers. This may also include economic provision for travel to an international laboratory within a scientific collaboration for the purpose of using a specific facility. | 0 |  |  |  |  |  |  |
| #3  BR  1.3 | Research should be consistent with international ethical standards. | An insistence on the highest principles of ethics and integrity underpins our research, and the welfare of research subjects is paramount. Our policies are designed to enable researchers to carry out their research effectively, whilst ensuring that it is consistent with the highest standards of quality, integrity and ethics. Evidence of thorough ethical review is a requirement of funding bodies across all disciplines, including the Research Councils.  The School of Medicine has its own School Research Ethics Committee which is responsible for ensuring that all non-clinical research carried out within the School has received the necessary ethical review and reports to the University Research Integrity and Ethics Committee; which has overarching responsibility for ensuring that research is conducted in accordance with the appropriate ethical, legal and professional frameworks, obligations and standards. These committees are held monthly and submissions are reviewed by a panel of experts.  Research involving NHS patients or relatives of patients must be approved by an NHS Research Ethics Committee | University Research Ethics Committee and structure ([www.liverpool.ac.uk/research-integrity/research-ethics](http://www.liverpool.ac.uk/research-integrity/research-ethics)) and also Safety Advisors Office ([www.liverpool.ac.uk/safety](http://www.liverpool.ac.uk/safety)). Implemented through local site safety committee (including biological safety) which includes PhD representative. | **Ethics in education and research:** The Faculty emphasises the importance of maintaining high ethical standards in all matters of education and research. More information about the Faculty’s commitment to upholding high ethical standards can be found on [our websites](http://www.uib.no/en/mofa/81596/ethics-research).  **Ethical training in the PhD programme:** In addition, the Faculty ensures that all PhD candidates are familiar with ethical requirements and principles, and that they are able to apply ethical considerations in their research. The PhD candidates are trained in research ethics through [MEDMET1 – Basic Course in Medical and Health Related Research](http://www.uib.no/en/course/MEDMET1). The course is mandatory to all PhD candidates at the Faculty. Furthermore, in accordance with the regulation of animal experimentation, § 13 Planning and performing experiments, the Faculty ensures that «all persons that plan or perform procedures in animals must receive a training program approved by the Norwegian food safety Authority. Our websites provides more information about [the Laboratory Animal Facility](http://www.uib.no/en/rg/animalfacility).  **Information and compliance with national regulations**: In order to ensure that The Faculty’s education and research complies with the national regulations on research ethics, all research projects must be considered for pre-approval by [The regional Committees for Medical and Health Research Ethics (REC)](https://helseforskning.etikkom.no/ikbViewer/page/forside?_ikbLanguageCode=us). As part of this, the Faculty puts great work into informing and advising the research environments about the existing regulations. [The Norwegian National Committees for Research Ethics.](https://www.etikkom.no/logg-inn?ReturnUrl=%2fen%2fIn-English%2f) Lastly, in 2010, the Faculty appointed an [Integrity Committee](http://www.uib.no/en/mofa/81597/integrity-committee-faculty-medicine-and-dentistry) as a resource for promoting ethical awareness among staff and students. | Control of the necessity for ethical permits for a given doctoral education project is made (1) at admission by the Departmental admissions board, (2) at halftime review by the review panel, and (3) upon application to defend the thesis by the KI dissertation committee.  A taught course in ethics (corresponding to 1.5 ECTS) is a compulsory training requirement for all doctoral students and must be completed within the first 2 years of study (see ‘General Syllabus for Doctoral Students’ page 4). | 0 |  |  |  |  |  |  |
| #4  BR  1.4 | There should be provision for allowing PhD candidates to perform part of their programme in another institution, national and abroad. | Cardiff University has an ambitious strategy to ensure it will consistently be amongst the top 100 universities in the world, recognised as an international university that is of benefit to Wales. Cardiff’s ambition to establish its position as a recognised global university will include the ongoing development of a diverse range of national and international partnerships that fall within the scope of collaborative provision. The term ‘collaborative provision’ describes a wide range of activity, involving various forms of collaborative and partnership arrangements both nationally and internationally. Such collaboration normally involves the provision of part, or all, of Cardiff University’s award through some form of activity with one or more ‘partner’ organisations or other bodies such as private companies. A network of support exists within the University, working actively to assist and provide guidance on collaborative proposals and helping to ensure that processes run efficiently and effectively, whilst also ensuring the right levels of scrutiny and rigour. A key consideration when approving collaborative activity is whether collaboration with a partner poses a threat to the University’s academic standards and student experience, and by implication the reputation of the University. The need to protect these is of paramount importance and must be the primary consideration in the evaluation of the benefits of any form of collaboration. Cardiff’s overarching Placement Learning Policy provides guidance for the development and management of placement provision. It covers all taught and research degree programmes undertaken in collaboration with employers and/or other organisations in Wales, the rest of the UK or internationally. Expenses may be provided by the project partners individually or collectively. A small number of students take advantage of these arrangements, the number is on the increase. | In principle all PhD students could have a placement but not all take one. Liverpool Doctoral College (LDC) offers 15 day placements outside academia with £500 bursary ([www.liverpool.ac.uk/study/post-graduate-research/liverpool-doctoral-college/placement-scheme/](http://www.liverpool.ac.uk/study/post-graduate-research/liverpool-doctoral-college/placement-scheme/)) .  Some PhD funding requires students to have a placement (e.g. BBSRC DTPs require 3 month PIPS; CASE studentships require 3 - 18 month industrial research placements; MSCA fellowships; collaboration with RIKEN (Japan) requires 2 years at RIKEN, funded by RIKEN).  Some research-related placements are funded by related research grants. | **Training abroad as part of the doctoral education:** The PhD programme at UiB is flexible with regards to studies abroad, and the Faculty strongly encourages all PhD candidates to spend time at a suitable institution abroad as part of their PhD degree. Establishing contact with suitable institutions and the planning of the studies abroad is normally done by the PhD candidate and his/her supervisor, with little involvement of the administration. If the candidate travels to a research institution abroad to learn techniques, conduct experiments or engage in other academic activities, the stay abroad may be approved as part of the training component's elective part. After returning, a report about the stay must be submitted (signed by the candidate and supervisor) in addition to a confirmation from the host institution regarding the duration of the stay and its content. PhD candidates who spend minimum 2 weeks at an institution abroad can apply to the Faculty for 1 ½ ECTS per week spent abroad. A maximum of 6 ECTS can be earned this way.  **Funding:** The Faculty of Medicine offers research scholarships for PhD candidates who plan to spend 1-6 months abroad as part of their PhD degree. PhD candidates are also encouraged to seek funding from other sources. Information about [relevant sources for additional funding is available online](http://www.uib.no/en/mofa/67056/financial-support-stays-abroad-employees-faculty). Since 2012 the Faculty has granted such research scholarships for research and/or studies abroad to 11 of our PhD candidates, for the total sum of NOK 619 375-,. Between two and three candidates have been granted research scholarships each semester, with the exception of 2014 where no candidates received scholarships. Scholarships ranges from NOK 25 000-, to NOK 101 000-,. All recipients of scholarships spent a minimum of two months abroad, and a majority of the recipients stayed four months or longer. All applicants who were granted scholarships received the full amount that they applied for.  **International engagement:** The Faculty is currently undergoing an evaluation of its own engagement in international cooperation through [the FRINDOC project](http://www.eua.be/activities-services/projects/current-projects/internationalisation/FRINDOC.aspx) | Study visits of variable time are recommended and frequently included in the individual study plan of many PhD students. These may be national or international. Specific international collaborative exchange PhD programmes currently exist with NIH and the Mayo Clinic (USA), Makerere University (Uganda) and in Singapore, as well as several EU-­‐funded ITNs which allow interaction with both academic institutions and industrial institutions.  Experience of more than a single research environment is thus highly recommended for all PhD students.  Such activities are common especially among preclinical PhD students. A maximum of 6 higher education credits is given for a period of research with a research group at a different university. | 0 |  |  |  |  |  |  |
| #5  QD  1.1 | Institutions lacking facilities/expertise could collaborate with stronger institutions to obtain these. | The quality and impact of Cardiff's research has led to a meteoric rise in league tables, pushing us into the UK's top 5 universities. We have broken into the "Golden Triangle" of Oxford, Cambridge and London, and confirmed our place as a world-leading university. We have climbed 17 places making us the fastest rising among the leading research universities of the Russell Group. Research by School of Medicine spans the spectrum from lab investigation to clinical practice, in hospital and community settings. From the discovery of a new bacteria resistant to antibiotics; creating new treatments for leukaemia, breast and prostate cancers; and improving dialysis treatment our world-leading research in clinical medicine has helped secure a GPA of 3.25 and a ranking of 8th in the UK. A distinctive feature of this unit is that 80% of our research has been deemed ‘outstanding’ for its impact in terms of its reach and significance. The Unit’s world-leading interdisciplinary research is ranked 4th in the UK with a GPA of 3.42. Impacts delivered through the research are far-reaching and varied in nature ranging from the introduction of new therapies and diagnostics, leading change in practice guidelines and standards, through to advancing and informing public-body policy initiatives. 100% of our research environment was rated as conducive to producing research of world-leading quality in terms of its vitality and sustainability. 90% of our research has been deemed ‘outstanding’ for its impact in terms of its reach and significance. In order to achieve these impressive results we collaborate with multiple institutions Internationally. | In IIB formal collaboration with RIKEN, A*STAR and Soleil where candidate spends 2 years at Liverpool and 2 at one of these research institutions. Doctoral Training Partnerships with Universities of Newcastle and Durham (BBSRC funding); Universities of Sheffield and York and Centres for Ecology and Hydrology (NERC funding) and Universities of Leeds, Liverpool, Newcastle and Sheffield (MRC funding). Many individual research collaborations of PhD supervisors to enhance research opportunities in their specific areas. | The Faculty collaborates with several institutions through different channels:  **National forums** for Vice Deans of research and other heads of PhD programmes (e.g., Head of Programme Boards etc.)  **National forums** for administrative staff for PhD programmes and doctoral educations  **Annual participation i**n the [Orpheus conference](http://www.orpheus-med.org/) and the [EUA-CDE meetings](http://www.eua.be/eua-cde-munich.aspx)  Regular contact with key partners, e.g., [Karolinska Institutet](http://ki.se/en/startpage), Sweden | Some forms of Intra-­‐ or Inter-­‐Departmental, Inter-­‐Campus or Inter-­‐ University (National or International) collaborations are typical of a KI PhD project.  It is expected that the required standards are already fulfilled by the host laboratory without the need of collaborative support. | 0 |  |  |  |  |  |  |
| #6  QD  1.2 | Possibilities for joint and double degrees could be explored. | Work in Progress | Yes. In IIB there is currently a joint/duel programme with one Thai University (no students at present, 3 in the past) | Yes. According to the PhD regulation’s §19, The institution may enter into a collaboration with one or more Norwegian or foreign institutions to collaborate on joint degrees (incl. cotutelle agreements.  In joint degrees, exceptions from the provisions can be made if necessitated by the collaborating institutions' regulations. Such exceptions must, both individually and jointly, be fully justifiable; see also § 19.4.  The standard process is in line with established rules as specified in the Regulations for the Philosophiae Doctor (PhD) degree at the University of Bergen. | A designated International Relations office at KI central administration explores these types of interactions, and several such exchange programmes are already established (e.g. with Makere University in Uganda). | 2 |  |  |  |  |  |  |
|  | | ***2. Outcomes** | | | | | |  |  |  |  |  |
| #7  BR  2.1 | PhD programmes should provide PhD candidates with competences to become qualified and independent researchers, according to principles of good research practice. | The Research Student Progress Monitoring Procedure provides a formal system of regular reporting and evaluation, which complements the continuous dialogue between the student and their supervisors, and the ongoing monitoring that is central to the supervision process. During the early stage of the research programme, the student and main supervisor are required to agree a Research Plan and to complete a Development Needs Analysis (i.e. a skills audit and plan). The Initial Reporting requires confirmation that these have been undertaken, and requires the student and main supervisor each to submit an Initial Report. The Initial Reporting is undertaken within the first three months of the student's registration. Completion of the Initial Reporting is endorsed by the School's Director of Postgraduate Research Studies. In addition, a Progress Report, the first of which normally to be completed within 9 months of registration (within 6 months of Initial Reporting) is completed every 6 months thereafter (normally at 9, 15, 21, 27, 33 months, etc.). The purpose of the Progress Report is to assess a student's progress with sufficient frequency to allow for remedial action to be taken if necessary. The process also involves reviewing and updating the Research Plan and so helps maintain the focus of the research, while re-evaluating the student's skills training needs. The exercise requires the production of two separate reports: the Student’s Self-Assessment Report recording the work done since the last Self-Assessment Report (or Initial Report) and the Supervisor’s Report commenting on progress. The reports are followed by a Progress Meeting of the student and supervisor. | Criteria are based on Vitae Researcher Development Framework ([www.vitae.ac.uk/researchers-professional-development/about-the-vitae-researcher-development-framework](http://www.vitae.ac.uk/researchers-professional-development/about-the-vitae-researcher-development-framework)), and Development Needs Analysis undertaken annually between PhD student and supervisor. Professional skill development is led by LDC Development Team ([www.liverpool.ac.uk/pgr-development](http://www.liverpool.ac.uk/pgr-development)) . | What sort of training is offered to make PhD candidates independent researchers? What training is provided for transferable skills? How are these competencies evaluated?  **Training**: Firstly, UiB follows the Salzburg principles of the third cycle within the Bologna process. Here, the 1st of the ten Salzburg principles may be of particular importance, as a core component of the doctoral training at the UiB is the advancement of knowledge through original research. Secondly, in accordance with the Bologna process, All candidates are required to complete a training component consisting of one semester’s fulltime studies, totalling 30 ECTS. As part of the training component, the PhD candidate completes both obligatory and elective courses. The authority to evaluate and approve elements in the training component lies with the Faculty. In some cases however, approval of elements in the training component is done by the Programme Board. It is also the Faculty that has the authority to approve the training component as a whole. The regulations for approval of elements in the training component are [available on our websites](http://www.uib.no/en/mofa/65665/regulations-and-guidelineshttp:/www.uib.no/en/mofa/65665/regulations-and-guidelines). Lastly, in accordance to the Bologna process, the Faculty and the Programme Board ensures that PhD activities comply with the current qualifications framework. This implies that the doctoral education must demonstrate a significantly higher level than that of the Master’s and Bachelor’s education.  **Evaluation:** The PhD candidates are evaluated consecutively during the stages of the doctoral education:   - Through annual progress reports (see point BS4.8) - Through the completion of [midway evaluation](http://www.uib.no/en/svf/48423/mid-way-evaluation), [FORMIDL901 – Scientific dissemination](http://www.uib.no/en/course/FORMIDL901), and [MEDMET1 – Basic Course in Medical and Health Related Research](http://www.uib.no/en/course/MEDMET1), all of which are included as obligatory elements in the training component - Through completion of elective courses, with an assessment upon completion of the course - Through participation in research schools, including seminars and network meetings. The meetings consist of weekly scientific presentations from various basic, translational or clinical PhD candidates, followed by discussions around the presentations. - Through participation in a research group, scientific network groups and during individual supervision sessions | Developing as an independent researcher is a basic Intended Learning Outcome (ILO) defined by the Swedish Higher Education Ordinance (see ‘Rules for Doctoral Education’ page 6), and is incorporated into every PhD student’s Individual study plan. Evaluation of progress to achievement of this aim is made at both halftime review and during thesis defence by dissertation committees.  An annual Departmental review allows for annual modifications of individual study plans, a primary cause being incorporation of new projects designed partly/wholly by the students themselves, indicating some degree of scientific independence.  PhD students have the possibility of taking part in various activities offered by the KI Careers service, the Doctoral Programmes, by the Board of Doctoral Education or by Departments themselves, including specific training of leadership, scientific commercialisation/patenting, pedagogic and teaching skills. Through these activities in combination with training in the generic skills such as scientific presentation, statistics, scientific writing, philosophy of science and research ethics, many of the attributes expected of an independent researcher can be developed. | 1 |  |  |  |  |  |  |
| #8  BR  2.2 | A PhD degree should also be of benefit in a career outside academic or clinical research (problem solving, analysis, evaluation, technology transfer etc.). | See above progress monitoring requirements and self-reflection on skills training (BR2.1).  The PGR Curriculum in the School of Medicine is centred upon four themes namely; Transition, Acquisition, Completion and Employment. Our students map their training requirements to each theme in order that their approach to training selection is balanced. | See BR2.1 above. Opportunities are provided and PhD candidates encouraged to take them up. There is an annual report on skills development undertaken by each to the university. There is no readily accessible record of training/development of these competencies and thus no formal audit to check candidates have taken opportunities. | Although most supervisors include career advice as part of supervision for the candidates, the Faculty does not yet have any formal mechanisms for providing our candidates with career advice. However, from autumn 2014 to spring 2015, the Faculty participated in a national working group appointed by the Norwegian Association of Higher Educations Institutions (UHR). The working group’s task was to formulate an inspirational report and by way of this report, contribute to the development of better and more systematic career politics among the higher education institutions. [The report is available online](http://www.google.no/url?sa=t&rct=j&q=&esrc=s&frm=1&source=web&cd=1&ved=0ahUKEwit8oXOqJ7JAhXHESwKHd6IDTgQFggbMAA&url=http%3A%2F%2Fwww.uhr.no%2Fdocuments%2F230615KarrieredokumentetUHR.pdf&usg=AFQjCNEJ4T4-0ctM-n0Os0lgDFqUxASdiA&bvm=bv.108194040,d.bGg) (NB! In Norwegian only) Central to the working group’s discussions and proposals are relevant processes at EU-level, especially [The European Charter for Researchers and the Code of Conduct for the Recruitment of Researchers](http://ec.europa.eu/euraxess/index.cfm/rights/europeanCharter). The UiB will build on the advices from this working group to develop a better and more systematic career politics for both PhD candidates and academic staff in all stages of their career. | Most of the skill sets described in BS2.1 are of use in careers within the pharmaceutical industry, within clinical posts, within non-­‐scientific appointments in society (e.g. as bank analysts, governmental investigators).  In a recent investigation of graduated alumni 88% stated that use of research skills were part of their current employment. | 0 |  |  |  |  |  |  |
| #9  BR  2.3 | The outcomes for PhD candidates with a background in medicine or other professional training are the same as for any other PhD. | All PGRs are managed identically. | IIB has PhD students with medicine and veterinarian degrees studying for PhD. The only special arrangement would be advice to attend undergraduate/masters modules (or more advanced training) if needed to provide science background for project (as advised to all IIB’s PhD candidates). The university also has an MD (Doctor of Medicine) degree for more medical-orientated research for those with a medical qualification but none are currently registered in IIB. | All PhD candidates are managed identically. (As for Cardiff) | All PhD candidates are managed identically. | 0 |  |  |  |  |  |  |
|  | | ***3. Admission policy and criteria** | | | | | |  |  |  |  | 0 |
| #10  BR  3.1 | PhD candidates should be selected on the basis of a competitive and transparent process. | Project calls are advertised nationally/internationally and PhD candidates are selected as follows:  Applications are made via the University’s online application service or directly to the supervisor. Candidates must submit  Supporting statement, CV, Qualification certificates, References x 2 and Proof of English language (if applicable)  The process for applying is made clear on the advertisements. The PGR Office is responsible for checking eligibility. Potential candidates must have obtained, or be about to obtain, a First or Upper Second Class UK Honours degree or the equivalent in an appropriate area of biomedical sciences. Applicants with a Lower Second Class degree will be considered if they also have a Master’s degree or have significant relevant non-academic experience. Candidates will need to have achieved at least 6.5 in IELTS (and no less than 6.5 in any section) by the start of the programme. A School Selection Panel shortlists a maximum of 6 potential candidates for interview on the basis of their anonymised supporting statement, CV and against the three additional criteria (Proven academic quality,  Research potential and Personal motivation). Main supervisors may contact the potential candidates to arrange an interview via video conference (e.g. Skype) in order to help with the shortlisting process. The shortlisted candidates to attend a face to face interview with the School Selection Panel. Interviews are normally 30 minutes in length and will include a 5 minute presentation (using no more than 3 slides). The Panel complete a standard template report for each candidate, which provides a numerical score against the stated selection criteria (e.g., written application and performance at interview). Shortlisted candidates will also meet the team that will host the candidature. Candidates benefit from face-to-face interaction in gaining an understanding of the project and the environment in which they will work. A candidate may formally commence his/her period of research on the first day of October, January, April or July, subject to the agreement of the Head of School concerned. | General process is that all projects are advertised on [www.FindAPhD.com](http://www.FindAPhD.com) , shortlisted by supervisor, interviewed (generally panel of 5) and offers of funded studentships or bursaries made, or the offer of a study position for self-funded candidates . Details can depend on source of funding (e.g. RCUK DTPs usually have member(s) of interview panel from outside IIB). Some sources of funding require a nominated candidate. Some students have their own funding (e.g. from overseas governments, universities) and in this case interview is to assess ability to undertake PhD-level research, rather than competition for funding. | The Faculty has two types of applicants: Applicants with funding through UiB fellowships, and applicants with funding from other sources than fellowships from the UiB.  **The process for applying for UiB fellowships**: The Faculty announces a few PhD fellowships each semester. The Faculty normally receive 80+ applications for a UiB fellowship each semester. When assessing potential candidates for UiB fellowships, the selection criteria are the same as those described in QD3.1. Based on an open and transparent process, between five and ten applicants are selected and offered a UiB fellowship.  **The application process for admission to the PhD programme^[[1]](#footnote-1)^**: Students when enrolling in the PhD programme need to have established contact with a member of the academic staff at UiB who is willing to act as supervisor. The application form should be filled out in cooperation with the supervisor, and should be sent by way of the department. A preliminary assessment of the application according to current rules is done by the department, prior to the application being forwarded to the Faculty. In the department’s evaluation of the applicant, three main principles acts as basis 1) the quality of the proposed PhD project, 2) the quality of the research environment in which the PhD project is included, and 3) the candidate’s merits and motivation. In addition to this, the department must evaluate if the proposed progress plan and plan for funding is realistic. On recommending admission, the department establishes that the relevant equipment and other infrastructure (office space, laboratory facilities etc.) are available for the candidate to complete the project in question.  It is the Faculty that makes the decision on admission. The admission decision is based on a comprehensive evaluation of the project description, the applicant's formal qualifications, sufficient resources for the realisation of the research project and the plan submitted for the research training, and is made on recommendation from the department in question. At least two supervisors must be appointed in the decision, responsibility for the handling of other needs outlined in the application must be allocated, and the agreement period/admission period must be set with a start and end date. The start date must correspond to the start date of the funding. The PhD candidate and the supervisors are informed of the result of the admission process by a letter as soon as a decision exists. Enclosed to the letter is also important information regarding the PhD programme and the department in question, as well the Regulations for the PhD programme at the Faculty (PhD Regulations) and the PhD agreement.  The PhD agreement states the rights and obligations of both parties and must be signed no later than one month after the candidate has been notified of admission. | From January 2014 onwards all doctoral positions at KI have to be advertised and selection made in competition.  In accordance with the Swedish Higher Education Ordinance, exceptions include:  (1) when admitting a doctoral student who is to complete the course or study programme within the framework of employment by an employer other than the higher education institution  (2) when admitting a doctoral student who has previously begun doctoral studies at another higher education institution, or  (3) if there are similar special grounds. | 1 |  |  |  |  |  |  |
| #11  BR  3.2 | Applicants for PhD programmes should have an educational level corresponding to a master’s degree. | Candidates must have obtained, or be about to obtain, a First or Upper Second Class UK Honours degree or the equivalent in an appropriate area of biomedical sciences. Applicants with a Lower Second Class degree will be considered if they also have a Master’s degree or have significant relevant non-academic experience. Candidates will need to have achieved at least 6.5 in IELTS (and no less than 6.5 in any section) by the start of the programme. A candidate for the degree of MD must have qualified for the degrees of Bachelor of Medicine and Bachelor of Surgery at least three years prior to admission. Irrespective of a candidate's qualifications, the School must satisfy itself that a candidate is of the required academic standard to complete the programme of research proposed. | Many have Masters, but not all. Medical and other professional degrees are accepted.  <https://www.liverpool.ac.uk/media/livacuk/tqsd/code-of-practice-on-assessment/appendix-1-PGR-CoP.pdf>  ’Applicants for postgraduate research study at Liverpool are normally expected to hold a UK first degree with a First Class or Upper Second Class degree classification, or a Second Class degree plus a Master’s degree. Equivalent international qualifications are also accepted, and their equivalence will be evaluated on the basis of the information provided by the National Academic Recognition and Information Centre (NARIC) as well as internal guidance based on our experience of a qualification’s suitability as a preparation for our programmes.’ | Describe the level required. Are persons with a medical degree or other professional degree accepted?  For admission to the PhD programme, the applicant must normally hold a five-year master's degree, with regards to the second cycle, or a cand.med. (6 years), cand.odont. (5 years), cand.pharm. (5 years), or cand.psychol.degree (6 years), or equivalent.  The Faculty may, following a separate assessment, approve another equivalent education as the basis for admission. The Faculty may stipulate further requirements to qualifications in supplementary regulations, following criteria that are publicly available and in line with the institution's recruitment policy and academic profile.  For education conducted outside of Norway, an approval from [the National Agency for Quality in Education (NOKUT)](http://www.nokut.no/en/) is required. | A person meets the general entry requirements for doctoral education if he or she:  has been awarded a degree at advanced (second-­‐cycle) level has satisfied the requirements for courses comprising at least 240 credits of which at least 60 credits were awarded at advanced level, or  has acquired substantially equivalent knowledge in some other way in Sweden or abroad.  Generally a master's degree is a *de facto* requirement.  The higher education institution may permit an exemption from the general entry requirements for an individual applicant, if there are special grounds.  [http://ki.se/ki/jsp/polopoly.jsp?l=en&d=29411&a=29596](http://ki.se/ki/jsp/polopoly.jsp?l=en&amp;d=29411&amp;a=29596) | 2 |  |  |  |  |  |  |
| #12  BR  3.3 | Before enrolment or at clearly defined times during the programme, the institution should evaluate and approve:  - Scientific quality of the project,  - Likelihood to complete within normal timeframe,  - The possibility for candidate to provide creative input,  - Qualifications of supervisors. | The Scientific quality of the project, supervisor track record, mentoring arrangements and compliance are assessed by an independent, expert panel assigned by each division. Approval for the potential candidature by the School is subject to satisfactory completion of the peer assessment procedure. Potential risks to on time & successful completion are identified and mitigated by contingency planning. Alternatively the project will not be approved for a potential candidature. The School has trained almost 200 of its supervisors over two years. This certification is necessary for our supervisors to recruit and constitutes a stop/go checkpoint for PGR provision in our School. For externally funded full time projects the creative input normally comes from the supervisor because they are subject to external peer review procedures prior to approval. For self-funded, international and staff candidates then the potential student will have creative input to the project. Our current on time submission rate for full time PhD students is 95% which evidences, testament to the appropriateness of our quality enhancement protocols. | Project approval depends on funding source. External funding organisation may select specific projects for funding. Where funding is via IIB, Institute’s senior management team will review PhD projects (and have some pre-set criteria). If candidate has own funding, supervisor with candidate can design project within funding available. Supervisors can advertise projects for self-funded students after approval by head of department. University requires annual review of progress of candidates to PhD, so including likelihood to complete within normal timeframe (3 - 4 years). | As stated in BS3.1., the Department’s evaluation of the PhD applicant rests on three main principles:   1. the quality of the proposed PhD project 2. The quality of the research environment behind the PhD project, including supervision of the PhD project. According to the Regulations §6.1, all supervisors must hold a PhD degree or equivalent. 3. the merits and motivation of the candidate   In addition to this, the department evaluates if the proposed progress plan and plan for funding is realistic.  In conclusion, the PhD project is approved before the assignment as a PhD candidate. | Admission of doctoral students is delegated to the Head of Department where the doctoral student will be registered. Each department has a Director of Doctoral Education who with a Departmental Admissions Board (comprising representative researchers and students) is responsible for preparing cases, organising and evaluating the admissions seminar, as well as submitting a proposal for a decision on admissions that serves as a guide for the Head of Department. Aside from the general and specific entry requirements, the individual study plan, funding plan, organisation and quality of supervision and of the research environment are all assessed for each case, and only those which are considered to promote doctoral education of the highest quality are recommended for admission.  An annual review is submitted to and reviewed by the Departmental Director of Doctoral Education, in which progress in achievement of intended learning outcomes, supervision, funding and scientific progress is assessed. Poor outcomes stimulate discussions with the Director, supervisors and students in order to strive for improvement.  A halftime review is conducted after 2-­‐years of full-­‐time equivalent study time. After proposal by the supervisor, the Head of Department will appoint a review committee consisting of three researchers with adequate knowledge of the subject, and who are independent from the project and have obtained a doctoral degree. A written report describing progress in learning and science is submitted and the student gives an open research seminar. (2017: A 10-page literature review is now also required). The board, together with the supervisors and the doctoral student, shall assess the prospects of the project leading to a doctoral degree, and propose any necessary changes to the individual study plan. In the case of insufficient quality at this point it is the duty of the review panel to clearly indicate this to the Departmental Director of Doctoral Education, who in turn must meet with student and supervisor to discuss a plan of action for improvement.  It is inherent in the Governmental Higher Education Ordinance that students should progress towards being independent scientists by providing input. | 0 |  |  |  |  |  |  |
| #13  BR  3.4 | A PhD programme should only be initiated when the resources for completion are available. | Financial support for the project is scrutinised prior to advertisement of any potential candidature and checked in advance of making a formal offer to a student. We have a two-stage procedure in the School where the finances are checked by the division that will host the candidature and then approved/declined by the Finance Director of the School subject to the information provided. | Full financing requires tuition fee, stipend and research costs for 4 years. Most projects are financed from outside the university. Specific studentship funding from e.g. UK government (RCUK DTPs), industry, charities generally provides full funding for UK/EU citizens. Internal IIB form is completed and reviewed before PhD study offer is made indicating source of finance. Parallel research grants can provide some research resources. | Describe how PhD programmes are financed and how it is ensured that full financing will be available.  Before admission, the applicants must document a plan for financial support. Admission depends on sufficient funding for the whole programme period (3 years), and must cover both expenses related to the PhD project as well as living costs. On recommending admission, the department establishes that the relevant equipment and other infrastructure (office space, laboratory facilities etc.) are available for the candidate to complete the project in question.  Most applicants have been granted national or international research fellowships to conduct their PhD project, or they have been released from other positions to work on their PhD thesis. In such cases, the Faculty requires that the applicant can document that they have been released from their current position for at least 20 % in order to work on their PhD thesis. | As a Swedish PhD encompasses a 4-­‐year period and as most research grants usually encompass 2-­‐ or 3-­‐year periods, then the economical, human and material resources are assessed at admission application by the Departmental Admissions Board and the Departmental Administration as a risk assessment. Based on the track record of the supervisor and the perceived possibility of maintaining resources required for the whole of the doctoral education period, the Department makes a decision to admit a student or not. In the event that resources should become limiting for some unexpected cause, the Department has responsibility for providing additional economic, human and material resources as required in order that a student can complete their studies within the 4-­‐year period | 0 |  |  |  |  |  |  |
| #14  QD  3.1 | In choosing PhD candidates, the applicants’ potential for research ought to be considered, not just past academic performance. | See  BR 3.2 and 3.3 | Where project funding is competitive between students and/or projects, CV (i.e. exam marks, research experience, references, letter of motivation) is used to shortlist and then selection made after an interview (in person or using Skype). Interview assesses ability to present and knowledge of research previously conducted; knowledge about proposed PhD project; evidence of teamworking, maths, IT and academic background. Formal marks can be given for each component to reach a decision. | In the application procedure, weight given to:  **Educational background**: The PhD applicant should have a GPA of A or B, and should have obtained the grade B or better on their master’s thesis (if applicable).  **Research experience**: Although extensive prior research experience is not a requirement, the applicant should have previous experience with conducting research.  **Expressed motivation:** The applicants motivation for the specific PhD project and for conducting a PhD degree is given great weight  **Performance at interview:** When establishing contact between the supervisor and applicant, the supervisor normally interviews several prospective candidates  **Letters of recommendation** from previous employers and/or supervisors  **The application should contain**: An extensive and detailed project description, including an academic outline of the project schedule, funding plan, documentation of specific requirements regarding academic and material resources, any plans for stays at another research institution (including abroad) or enterprise, a plan for academic dissemination, details of any intellectual property restrictions to protect the rights of other, a plan for the training component, including training that will provide a general competence in keeping with the qualifications framework, a proposal for at least one supervisor and an indication of affiliation with an active research community, a description of any legal and ethical issues raised by the project and how these can be resolved.  The academic environment the PhD candidate is to be affiliated with should actively participate in developing the project description and in the programme for the actual PhD education. | Selection of PhD students is delegated to the supervisors that will be involved in the research project. Criteria for selection are therefore decided upon by the individual. See Rules for Doctoral Education Section 2.3:  Selection from amongst the applicants will take place on the following grounds:   - documented knowledge of the subject that is of significance to the research area - analytical expertise - other documented knowledge/experience which may be of significance for doctoral education in the subject.   A combined assessment of the applicants’ qualifications and suitability will be conducted. | 0 |  |  |  |  |  |  |
| #15  QD  3.2 | Projects ought to be externally assessed by written project description or presentation to panel of independent scientists. | External experts may be recruited from Industry, Government or other Higher Education Institutions. All studentship funding obtained from UK research charities is achieved by success in open competition. | Yes, for RCUK DTPs PhD projects are assessed by the partnership (scored by up to 12 people on series of formal criteria). For International partnerships project are reviewed by Institute and also by Faculty prior to decision to allow project to be advertised. External funders (charities, industry, RCUK) usually select which projects they fund. See also BR3.3 and BR3.4. | Not all PhD project are externally assessed. However, PhD projects with funding from external sources (e.g. EU funding, The Research Council of Norway or the Western Norway Regional Health Authority, etc.) will be assessed by the external source of funding during the application process for such funds. Research projects with funding from the UiB will undergo a prior assessment from an independent scientific committee. The assessment committee consists of one representative from each of the five departments, as well as the Heads of Research from each department.  In addition, the specific PhD project will also be internally evaluated at the department level as part of the individual PhD candidate’s application for admission to the PhD programme. | The Admissions Board within each Department is appointed by the Department and includes the Departmental Director of Doctoral Education, at least one student representative and at least 3 senior scientists with subject-­‐specific competences to cover areas of study within the Department (large Departments have larger Admissions Boards).  In case of lack of specific subject-­‐specific competence within a Department then appropriately competent scientists from other Departments (or externally of KI) are identified and asked to review the research plan. | 0 |  |  |  |  |  |  |
| #16  QD  3.3 | If the PhD candidate is obliged to obtain extra income, it ought to be ensured that the PhD candidate has the necessary time to complete the programme. | PhD candidates obliged to obtain extra income are rarely registered full time (if at all) in order to ensure that the PhD candidate has the necessary time to complete the programme on time. | University policy is to allow up to 15 h work per week for full-time registration (so allowing some teaching duties). Part-time registration (max 6 years) is available to UK/EU citizens who have to work. | It is not uncommon that PhD candidates, and especially candidates which undergo clinical/professional work parallel to their doctoral education, apply for part-time work with their PhD, and as stated in BS4.7, several of our PhD candidates have been admitted to the PhD programme based on a plan of reduced progression with their PhD degree due to teaching or clinical duties. Is stated previously, in such cases, the PhD period is extended according to the indicated progress (at least 50 % progress is required at the point of admission). | Parallel activities can be defined up to a maximum activity of 50%, but the individual study plan timeline reflects only time spent during doctoral education and has to reflect the requirement of 4-­‐years full-­‐time equivalent time spent with the doctoral studies. | 0 |  |  |  |  |  |  |
|  | | ***4. PhD training programme** | | | | | |  |  |  |  | 2 |
| #17  BR  4.1 | Programmes should be based on original research, courses and other activities which include analytical and critical thinking. | The training provided enables our PGRs to meet the requirements of the Framework for Higher Education Qualifications in England, Wales and Northern Ireland (Level 8 descriptor) in full. PGR provision in the School supports the creation and interpretation of new knowledge, through original research, of a quality to satisfy peer review, extend the forefront of the discipline and merit publication. By the end of their training our PGRs are typically able to make informed judgements on complex issues in specialist fields and are able to communicate their ideas and conclusions effectively to specialist and non-specialist audiences. Our programme equips our PGRs with the qualities and transferable skills necessary for employment (100% of our PGRs are employed within 6 months of completion). Our bespoke training is complemented by the excellent training and skills development programme delivered by The Doctoral Academy at Cardiff University. The Doctoral Academy works to strengthen the research community by promoting and supporting the delivery of an outstanding student experience, including a comprehensive training programme.  <https://www.cardiff.ac.uk/study/postgraduate/research/doctoral-academy>  Integrated Doctoral Programmes are defined as 4-year programmes that include a separate and structured element of preparatory research skills and methods training embedded within the first year of the PhD registration. | Research project is required, meeting e.g. QAA precepts for research degree (see <https://www.liverpool.ac.uk/media/livacuk/tqsd/code-of-practice-on-assessment/PGR-code-of-practice.pdf>)  Candidates have to also undertake any mandatory (e.g. safety) and departmental training. In IIB we require candidates to take 3 M-level modules (or equivalent e.g. languages) unless candidates already have a relevant Masters degree or we cannot offer anything relevant. External advanced training is encouraged. Supervisors generally organise journal clubs which help with analytical and critical thinking.  See also BR2.1, 2.2 | According to the Regulations for the Philosophiae Doctor (PhD) degree at the University of Bergen, with supplementary regulations for The Faculty of Medicine (hereafter referred to as the PhD regulations), § 2.2., the content of the PhD training is as follows:  The training includes independent research which must be documented by an academic thesis of an international standard at a high academic level. Additionally, the PhD candidate must undergo a training component, providing training in the disciplinary context, methods and theories that provide a disciplinary breadth and depth in their field, and that also contextualises the discipline within a broader framework. The PhD training is to train PhD candidates in the dissemination of academic work to the scientific community, colleagues, students and the general public.  Reference is here made to the Norwegian Qualifications Framework. The Norwegian Qualifications Framework is adapted to the European qualifications framework for higher education. The Framework describes qualifications in higher education through learning outcomes, rather than input. For each of the three main levels of higher education (Bachelor’s, Master’s and PhD), learning outcome is divided into knowledge, skills and general competence. More information about the expected learning outcome at each level of higher education can be found in [the Norwegian Qualifications Framework. Levels and learning outcome descriptors](file:///C:\Users\au184\AppData\Local\Microsoft\Windows\INetCache\Content.Outlook\0EYU0S41\Attachment%204%20NQF%20Higher%20education%20(BS4.1).pdf). | Each individual study plan is meticulously prepared for each PhD student. Apart from details about specific input from all supervisors, time and economic plans, there is a suggested project plan for a 4-­‐year period with described personalised ILOs, as well as definition of all taught courses, journal/book club, conferences and study visits that encompass the different learning activities that are expected to allow for development of a critical scientific approach. | 0 |  |  |  |  |  |  |
| #18  BR  4.2 | Programmes should be performed under structured supervision. | Each research student must have a supervisory team comprising at least two academic supervisors. The supervisory team may include additional academic and/or professional supervisors, and/or post-doctoral researchers. All must have a clear understanding of their role and responsibilities, which is also shared by the student. In all cases, the primary point of contact must be clear to the student, and an alternative contact if the main supervisor is not available. The Head of School (or nominees) namely the Director of Postgraduate Research studies is responsible for the appointment of staff as supervisors to the research students enrolled in their School, and for ensuring the continuation of regular and appropriate supervision throughout each candidature. To be appointed to a supervisory team, a supervisor should be experienced in research and possess an appropriate understanding of the proposed project to be able to make a positive contribution. At least one member of the supervisory team, typically a main supervisor, should have supported at least one successful completion of the relevant research degree and be currently engaged in high-quality research in the discipline. The School provides the student with a regular opportunity to comment on their satisfaction with their supervisory arrangements, typically coinciding with the Six-Monthly reporting cycle that forms part of the Research Student Progress Monitoring Procedure. A supervisory meeting is considered to be a formal, uninterrupted event, to which sufficient time and attention should be devoted. Both the student and supervisor(s) should be clear on the purpose of the meetings and keep agreed, documented records of the outcomes. The expected frequency and format of scheduled meetings with both the main supervisor(s) and with member(s) of the wider supervisory team should be agreed at the outset and adjusted, as appropriate, as the project progresses. | Candidates must have at least 2 supervisors, appointed when study offer is made. University requires at least 1 supervisory meeting recorded electronically each month (full-time PhD) or 1 every 2 months (part-time PhD). In practice, many students meet supervisors daily and use supervisory meetings for more formal reviews of progress. | Before admission, applicants for the PhD programme must establish contact with a member of the scientific staff at UiB who is willing to act as supervisor. The PhD regulations §6.1, state the following requirements for supervisors:   1. Supervisors must hold a PhD degree or equivalent; 2. Supervisors must be an active researcher, and should be part of a scientific environment of a high standard, preferably in the frontline of the research area 3. At least one of the appointed supervisors should have previous experience of supervision of candidates at the master's and/or PhD level.   The supervisors are appointed by the Faculty and a principal supervisor is formally appointed at the time of admission. | At least two supervisors are appointed for each PhD student  at least one of the supervisors must be an Associate Professor or a professor  the principal supervisor (under normal circumstances) must be active at KI  at least one of the supervisors, preferably the principal supervisor, must be active in the department to which the doctoral student is admitted.  A doctoral degree is required for a person to be appointed as supervisor.  All supervisors shall be thoroughly familiar with KI's regulations governing doctoral education, and a mandatory supervisor training course must be conducted before becoming a principal supervisor for all new supervisors.  A web-­‐based course must be conducted by all senior supervisors, as well as supervisors from other Universities.  In addition, other PhD students, postdocs or technical staff within a specific research group may give the PhD student daily support | 0 |  |  |  |  |  |  |
| #19  BR  4.3 | Programmes should ensure that PhD candidates have appropriate training in ethics and responsible conduct of research. | The University requires that all its researchers maintain exemplary standards of professionalism, academic practice, rigour and honesty in every aspect of research. To support this, the University has in place a Research Integrity & Governance Code of Practice and associated research ethics guidelines, which take into account policies and guidance originating from UK and international agencies, funding bodies and professional societies. The Research Integrity & Governance Code of Practice and associated guidelines apply to all research carried out under the auspices of the University, including that by students. All students engaged in research should familiarise themselves with the Research Integrity & Governance Code of Practice and the Student Guide to Academic Integrity and observe the relevant provisions they contain. Formal training is provided by the School at Induction upon initiation of the candidature via an interactive workshop. Responsibility for research conduct and integrity in their research degree programme resides with the student, with guidance from their supervisors. Additional support is provided through training offered by the Doctoral Academy. Ethical issues are overseen by the University Research Ethics Committee, supported by a network of designated Ethics Officers. The School is obliged to consider the nature of potential ethical issues in the conduct of its research, ensuring that appropriate records of projects, decisions and practices are kept, including those related to students' research. | All must attend an Academic Integrity lecture if attending or teaching on modules. Those working with animals must attend specific training and obtain relevant personal licence. Those working with other controlled organisms (e.g. GMOs, pathogens) must have relevant approval and work plans. LDC includes research ethics topics in the 3 Postgraduate Researcher Weeks each year (e.g. A brief introduction to Open Access and Research Data Management ). The HLS Faculty is currently developing its own training portfolio and a requirement for all candidates to attend relevant ethics training is being proposed. | According to the Guidelines for calculating course credits in the PhD programme, all PhD candidates are required to take one of the following courses in ethics and responsible conduct of research:  [MEDMET1 – Basic Course in Medical and Health Related Research](http://www.uib.no/en/course/MEDMET1) (8 ECTS)  or  [INTH301 – Basic Course in Research Tools and Theory](http://www.uib.no/emne/INTH301) (6 ECTS)  Both courses include topics in the philosophy of science, research ethics, publications ethics and scientific methods. Equivalent courses from other institutions may be approved on application.  Furthermore, the guidelines also state that candidates whose research project includes the use of laboratory animals, training in the use of laboratory animals in medical research (LAS201 and LAS202 or LAS203) is mandatory. Equivalent courses from other institutions may be approved on application.  [LAS201 – Animal Science Course](http://www.uib.no/en/course/LAS201) (2 ECTS)  and  [LAS202 – Mammal Science Course](http://www.uib.no/en/course/LAS202) (6 ECTS)  or  [LAS203 – Fish Science Course](http://www.uib.no/en/course/LAS203) (6 ECTS) | There is a formal requirement for attendance of a research ethics course as part of the Basic Course (ECTS) requirement, encompassing both research ethics concerning humans and animals as well as the ethics of correct research conduct and publication practices.  In addition, PhD students are expected to participate in preparation of ethical permits associated with their PhD projects. | 0 |  |  |  |  |  |  |
| #20  BR  4.4 | Programmes should have clear 3-4 year timeframe. Extensions should be possible but limited and exceptional. | The minimum period of study by pursuing a PhD candidature full-time in the University, or full-time in an external place of employment, or as a member of staff is three years (the first year is regarded as probationary). A candidature for PhD shall lapse if a thesis is not submitted, in the form and manner prescribed by the University, by the following time limit for completion: four years from the beginning of the candidate's period of study. Our average time to completion is 3.6 years in the School of Medicine. A period of study may be interrupted or a time limit extended by the University in accordance with the Student Attendance and Engagement Procedures and/or Extension to Time Limit Procedure – Postgraduate Research Candidates. A student's application for an extension to their latest submission date may be considered on one or more of the following grounds: ill health,  compassionate grounds, maternity/paternity leave, parental leave, exceptional professional commitments or unavoidable practical or logistical difficulties. | Normally it is 4 years to submission for more than 85% full-time students in IIB. A very few students submit after 3 years, most from 3.5 - 4 years with majority at 3 years 11 months. (Part-time PhD students have maximum 7 years, reduced to 6 since 2015. The number of part-time students is very small (5 currently) but most complete within the time allowed).  Current Ordinance on PhD length is 57A is <https://www.liverpool.ac.uk/corporate-governance-and-support-office/how-we-are-governed/programme-ordinances/>  Policy on suspensions and extensions is <https://www.liverpool.ac.uk/media/livacuk/tqsd/code-of-practice-on-assessment/appendix-6-PGR-CoP.pdf> Candidates apply via an on-line form process, generally within 2 months of expected completion date, requiring reasons, a thesis completion timetable and support from supervisor and Institute Director of Postgraduate Research in making case to Faculty and University administration. | **Standard length of PhD programmes:** According to the PhD regulations §5.4, the standard length of the PhD training is three (3) years of full-time studies. PhD candidates who are employed through a UiB research fellowship, obligatory work accounts for 25 % of the fixed term of employment. Due to this, the standard length of the PhD education for UiB research fellows is four (4) years of full-time studies. Furthermore, the PhD regulations §5.4 states that it is not acceptable to plan to complete the PhD education at a rate of progress that leads to a course of study that is longer than six (6) years. The maximum duration of a PhD programme is normally eight (8) years from the start date, not including statutory leave and required duties. The PhD period can be shorter than three years (full time), if the candidate has already completed parts of his/her research training, or if the admission is based on previous employment in a recruitment position (PhD position, research assistant or similar positions), meaning that the total time for the research training project adds up to three years. Statistics show that the current situation at the Faculty is in line with the established rules. The average PhD period for all candidates at the Faculty was 3.8 years (2014). The average PhD period only for candidates at the Faculty with employment through a UiB research fellowship was 4.0 years (2014) ^[[2]](#footnote-2)^.  **Extensions:** The agreement period may be extended by leaves of absence granted in accordance with the candidate's rights as an employee, or in accordance with rights granted by other funding sources. In the event of statutory interruptions, the admission period is extended correspondingly. On application, the admission period may also be extended on other grounds. The application must include an explanation of what has been accomplished/ published and what remains of the work towards the PhD degree. Applications for an extension may be approved if the Faculty, following a comprehensive assessment, determines that completion of the project is feasible within the extended period. An affirmation from the supervisor and the basic academic unit about supervision during the period of extension must be presented. In practice, it is not uncommon that PhD candidates apply for, and are granted extensions. Included extensions, the average PhD period for all PhD candidates at the Faculty was 4.7 years (2014). The average PhD period only for PhD candidates at the Faculty with employment through a UiB research fellowship was 4.6 years (2014)^[[3]](#footnote-3)^. | 4-­‐years equivalent full-­‐time study (8 semesters of 100% activity) is expected. While a sufficient number of scientific papers could be produced within a shorter timeframe, the scientific maturity expected of graduating students is expected to be first developed after 3 years.  Extensions are possible. The average time to completion is 4.5 years. | 0 |  |  |  |  |  |  |
| #21  BR  4.5 | Programmes should include relevant activities not directly related to the project (e.g. courses, journal clubs, participation in conferences, seminars and workshops, including preparation time) totalling about 15% of the whole programme. A substantial part should be concerned with training in transferable skills. (NB. “training” can be liberally interpreted as all scientific activities not directly related to the project, e.g. journal clubs, conferences, etc.). | The following are reasonable expectations for a student to have in relation to support for their research degree programme:  1. access to induction and information that promote an understanding of the academic environment in which their research studies will be undertaken;  2. access to a learning infrastructure and research environment that supports the progress of their research studies: this may sometimes be provided through collaboration with other institutions or partners;  3. a challenging and realistic project, with high-quality research training;  4. a supervisory team with the necessary expertise and experience, that will provide appropriate direction and guidance;  5. an adequate schedule of formal contacts and meetings with their main supervisor and their supervisory team;  6. honest and constructive feedback from supervisors and reviewers, and effective monitoring of academic progress;  7. the return of written work, with appropriately detailed feedback, within an agreed time scale;  8. responses to queries they raise with their supervisors within a reasonable time scale;  9. opportunities to develop broader research and professional skills;  10. opportunities and encouragement to engage with other researchers, and to present and discuss their research;  11. where feasible, opportunities to participate in teaching activities and access to appropriate training and mentoring arrangements.  Assessment of training needs/engagement is made formally at each Progress Monitoring event. | The University currently does not have a central, accessible record of training, conferences etc attended by each candidate. LDC Development (<https://www.liverpool.ac.uk/intranet/doctoral-college/development/>) oversee university level training opportunities. See training catalogue at <https://www.liverpool.ac.uk/intranet/doctoral-college/development/catalogue/>.  Health and Life Sciences Faculty is currently developing its own training portfolio based on what is provided by each Institute.  There is no formal assessment (unless students are enrolled for undergrad or Masters modules). Candidates have to make an annual report to the University of their skills training, conference attendance and other activities via a Portfolio of Activities.  See also BR4.1 | **Courses:** For a list of available courses and their evaluation forms, please consult [the Faculty’s overview of taught courses](http://www.uib.no/mofa/emner).    **Research schools:** Upon admission, PhD candidates are also linked to relevant research schools. The research schools arrange a great variety of activities such as weekly seminars and network meetings, research courses and training – all of which PhD candidates participate actively in. All research schools offer special courses to graduate students, PhD candidates and researchers. Information about all [research schools affiliated with the Faculty](http://www.uib.no/en/mofa/63870/research-schools) is available on our websites. All active research schools undergo a yearly review by the Programme Board of PhD programme.  **Assessment:** Course assessment must include the following:   - Evaluations of the course from students, preferably as assessment of the teaching. A summary of the student evaluations is to be published through [Database for quality assurance reports](https://quality.app.uib.no/). - Assessment of whether the progress and organising of the course confers with the established goals for the course, comments to the student evaluations, and other forms of evaluation. In addition - Comments of previous work with evaluations and the follow-up of such evaluations. - At least 1/3 of all taught courses must be evaluated each year. | Corresponding to 30 ECTS are required for a PhD (20 weeks): Basic courses – corresponding to at least 7.5 ECTS  Subject-­‐specific courses – corresponding to at least 9 ECTS  Conferences, teaching, study visits, journal clubs – corresponding to at least 4.5 ECTS  The remaining credits can be achieved through additional subject-­‐specific or non subject-­‐specific courses (e.g. about scientific innovation in the pharmaceutical industry, introduction to teaching), teaching activities, presentations at international conferences, research visits abroad) in order to satisfy the individual’s future career requirements.  Generic skills are included in the basic courses, and these must be completed prior to the halftime review.  See the General syllabus: https://internwebben.ki.se/sites/default/files/allman_studieplan_eng_faststalld.pdf | 2 |  |  |  |  |  |  |
| #22  BR  4.6 | PhD programmes that are performed in parallel with clinical or other professional training should have the same time for research and course work as any other PhD. | The School requires formal confirmation from the service provider that the potential candidate be permitted time out of programme to conduct their research project and receive appropriate training. The School must be assured that he potential candidate will not be disadvantaged in any way by their professional commitments and that all reasonable step have been taken to ‘protect’ research time for that individual. | We do not have this situation in IIB. University has only recently permitted students to be registered for 2 degrees simultaneously. Registering as a part-time student is probably the solution to give enough time, or taking the MD rather than PhD degree. | Yes. | The requirements outlined in BS4.5 and all general requirements are the same for clinical and preclinical PhD students.  Many clinical students attend defined clinical ‘Research Schools’ in which the course activities are organised for a specific cohort of students, ensuring them approval of time away from their clinical duties.  A minimum of 50% defined time for doctoral education is a prerequisite for admission of a clinical PhD student, equating to 8 years accumulated time. | 2 |  |  |  |  |  |  |
| #23  BR  4.7 | Progress of PhD candidates should be continuously assessed by the institution throughout the PhD. | In addition to BR 2.1, The progress of every research student is formally reviewed on an annual basis up until the successful submission of the thesis. The first Formal Progress Review is normally completed within 9 months of the student first  registering, and at 12-monthly intervals thereafter (so normally completed within 9, 21, 33 months). The School's arrangements for conducting the Annual Formal Progress Review and the criteria for proceeding are clearly communicated to our students and staff. Each Annual Formal Progress Review contains the following elements:   1. a substantial piece of written work from the student, to be considered by a Review Panel; 2. a meeting of the student and the Review Panel; 3. a documented outcome of the review and a recommendation.   The student is required to attend the Review Panel meeting set up to consider his/her progress. Following the meeting, the Review Panel will make one of the following  Recommendations to the School/University:   1. that the student's progress and standard of work is satisfactory and that the student is likely to complete their degree successfully and on timescale; 2. the student’s progress and standard of work is satisfactory overall but that one or more unsatisfactory aspects of progress and/or performance have been identified 3. that the student's progress and/or standard of work is unsatisfactory in which case he/she may be required to change his/her registration from a doctoral degree to MPhil OR he/she may be placed on a Warning of Exclusion from Research Study | Each student is assessed annually by the University using an on-line form, and in the Institute through provision of a report or plan which is then discussed with two assessors (independent progress assessment panel, IPAP) who provide a report, focusing on whether adequate progress to PhD thesis has been made. See <https://www.liverpool.ac.uk/media/livacuk/tqsd/code-of-practice-on-assessment/appendix-3-PGR-CoP.pdf> | **Annual progress reports/regular reporting**: According to the PhD regulations §9, all PhD candidates and principal supervisors in the PhD programme are committed to give annual reports regarding the progress of PhD degree with regards to the PhD project and the training component. The candidate and supervisors each submit a written report regarding the progress of the PhD programme. The reports are sent to, and approved by, the Faculty.  **Midway evaluations:** According to the PhD regulations §6.3 and Guidelines for calculating course credits in the PhD programme each PhD candidate must undergo a midway assessment. As a main rule, the midway assessment will include academic input from researchers within the PhD candidate's field and/or related fields. As with the regular reporting, the purpose of the midway assessment is to help the PhD candidate by identifying issues that entail a risk of delaying the project or bringing it to a halt, and to offer input that improves the quality of the work. The Faculty, supervisor(s) and the PhD candidate are required to follow up actively on issues that entail a risk of delays or of a failure to complete the PhD education, to ensure that the programme as far as possible can be completed within the nominal timeframe.  In addition to any regulated forms of evaluation, PhD candidates are consecutively provided with feedback from other PhD candidates and the respective research environment through their participation in research schools. | There are multiple points of evaluation and quality control, as described in BS3.3. | 0 |  |  |  |  |  |  |
| #24  QD  4.1 | For PhDs performed by clinicians, leave-of-absence from clinical duties could be provided for the PhD part of such programmes | Students are expected to pursue their research degree programmes on a continuous basis. It is recognised, however, that students may encounter circumstances that seriously disrupt or delay their research, and an Interruption of Study may be permitted if a student needs to suspend their research studies due to circumstances largely beyond their control. | It can be. Funding for full-time PhD study by clinicians is available. | Yes. It is common for clinicians to propose educational plans which involve part-time work with the PhD thesis, and part-time clinical duties. The estimated PhD period for such candidates is normally six (6) years. | Every clinical departmental head has to sign the individual study plan for each clinical PhD student, guaranteeing the minimum leave of absence required for enrolment in a PhD programme, which is 50%. Clinical PhD students are recommended to apply to the various clinical research schools in which cohorts of students receive the educational activities together at defined times, in which case their participation is guaranteed by the host clinic. | 0 |  |  |  |  |  |  |
| #25  QD  4.2 | PhD programmes could where relevant have an element of interdisciplinarity. | Interdisciplinary research is positively encouraged. | Many, depending on how broadly interdisciplinary is defined. Examples: using mass spectrometry of peptides to assess animal behaviour; the dynamics of helminth-virus interactions in the wild including mathematical modelling; business studies and applications practical of algae for entrepreneurship; Computational and mathematical approaches for proteomics - working with Big Data in biological and clinical research; A genomic approach to understanding crude oil recovery; Modularity and Synthetic Engineering of carbon-fixing organelles | Interdisciplinary research is positively encouraged. As an example, the Faculty of Medicine actively cooperates with other Faculties and external institutions regarding project development, funding and supervision of PhD candidates. | Interdisciplinary research is positively encouraged and very common. | 0 |  |  |  |  |  |  |
|  | | ***5. Supervision** | | | | | |  |  |  |  | 0 |
| #26  BR  5.1 | Each PhD candidate should have a principal supervisor and normally at least one co-supervisor. | For each candidature, the Head of School concerned shall appoint at least one main supervisor who is a member of staff employed in that School. Staff appointed as additional supervisors by the Head of School may be external to the School concerned or to the University. Each research student must have a supervisory team comprising at least two academic supervisors. The arrangement normally fits one of two models below:   1. Main supervisor plus second supervisor(s) where the subject expertise is substantially or primarily provided by one supervisor: at the very least, a second supervisor provides an additional contact to whom the student can discuss general academic issues, and provides input as part of the Six-Monthly reporting cycle. 2. Joint supervision where, for example, the research project is interdisciplinary, involves collaboration with a partner university or research organisation, or where an inexperienced supervisor is being supported by an experienced colleague: both joint supervisors provide expert guidance to the student, but one is designated as the principal supervisor, assumes overall responsibility for the management of the student, and is recognised as their first point of contact; further supervisory team members may also be appointed. | At least 2. Percentage split between them (90/10, 50/500 indicates relative roles. Primary supervisor takes lead on project. See <https://www.liverpool.ac.uk/media/livacuk/tqsd/code-of-practice-on-assessment/appendix-2-PGR-CoP.pdf> | The PhD regulations §6.1, state that all candidates who are admitted to the PhD programme at The Faculty of Medicine, must have at least two supervisors, whereof at least one of the supervisors must be affiliated with the candidate’s department. It is not recommended to have more than four supervisors. In practice, more than 75 % of the PhD candidates have either two or three candidates. | All students must have a principal supervisor and at least 1 co-­‐supervisor, although more than 4 supervisors in total is generally not recommended.  All students must have an external mentor that is independent from the research project. See BS4.2. | 0 |  |  |  |  |  |  |
| #27  BR  5.2 | The number of PhD candidates per supervisor should be compatible with the supervisor’s workload. | As a guide, an individual member of staff should not normally, at any one time, be supervising more than six students as a main (or 'joint-main') supervisor. It is recognised, however, that some supervisors may have the capacity to supervise a higher numbers of students effectively, depending on their individual commitments and the nature of the research environment in which they work. A supervisor in the School will usually be supervising 3 to 4 students in total. | In IIB 1 - 3 is normal. 0 – 6 is the range. | The PhD regulations §6.1, state that each PhD candidate must have at least two supervisors, one of whom should be appointed as the principal supervisor. The number of PhD candidates per supervisor ranges from one to 14 per supervisor (including both principal and co-supervision). However, a majority of supervisors are principal supervisor and/or co-supervisor to no more than three PhD candidates. More than 80 % are principal supervisor to one or two candidates. | Assessment of the academic, social and leadership skills of supervisors, as well as their perceived availability to efficiently supervise, is made by the Departmental Director of Doctoral Education and the other members of the Admission Board at the time of admission application. It is the Head of Department who takes the final decision concerning admissions.  It is the duty of the Director to restrict (or prevent) the number of PhD students allowed to be supervised by a given supervisor.  In one Department (Clinical Neuroscience) all potential supervisors must be approved by the Department annually before they can even be considered. (2017: Mandatory for all Departments) | 0 |  |  |  |  |  |  |
| #28  BR  5.3 | Supervisors should be scientifically qualified and active scholars in the field concerned. | To be appointed to a supervisory team, a supervisor should: .   1. Have a research degree (MD/PhD) 2. be experienced in research; 3. possess an appropriate understanding of the proposed project to be able to make a positive contribution. 4. At least one member of the supervisory team, typically a main supervisor, should have supported at least one successful completion of the relevant research degree; 5. be currently engaged in high-quality research in the discipline | All tenured and tenure-track members of staff can be supervisors. At least one must have supervised a PhD to completion. See <https://www.liverpool.ac.uk/media/livacuk/tqsd/code-of-practice-on-assessment/appendix-2-PGR-CoP.pdf> Information on staff and qualifications: <https://www.liverpool.ac.uk/integrative-biology/staff/> | According to the PhD regulations, §6.1, the supervisor(s) must have a doctoral degree or equivalent academic competence within the subject area, and be an active researcher. At least one of the appointed supervisors should have previous experience of supervision of candidates at the master's and/or PhD level. | The supervisory team (principal and co-­‐supervisors) must encompass sufficient scientific knowledge within the whole subject of study for an individual PhD student. Different supervisors may thus contribute with different, complementary competences.  Perceived scientific track record of scientific quality is inherent in review of supervisor suitability at admissions procedure.  One of the supervisors must be a Professor or Associate Professor. | 0 |  |  |  |  |  |  |
| #29  BR  5.4 | Supervisors should have regular consultations with their PhD candidates. | The School expects that:  The student will meet formally with their main supervisor(s) at the commencement of their programme. Thereafter, the student and their supervisors will then meet in accordance with an agreed frequency of formal meetings. Although the frequency may vary (between disciplines and mode of study, according to the relative development and needs of the student, and at different stages of the research project), formal meetings should generally take place between student and main supervisor(s) on at least a monthly basis, in person or by video chat. .The schedule of supervisory meetings will be integrated with the requirements of the Research Student Progress Monitoring Procedure. | Required to record 12/6 supervisory meetings annually full-time/part-time. In practice most supervisors have ‘open door’ policy and regular group meetings so meet much more frequently.  See Section 3 about supervisory meetings <https://www.liverpool.ac.uk/media/livacuk/tqsd/code-of-practice-on-assessment/appendix-3-PGR-CoP.pdf> | The PhD regulation, §6.2, state that the candidate and supervisor should be in regular contact. Beyond this, neither the PhD regulations nor other guidelines specify the frequency of consultations between PhD candidates and their supervisors. However, PhD candidates and supervisors are expected to meet several times per month to discuss the progress of the project. In addition to this, the research groups and research schools ensures regular contact as both PhD candidates and supervisors participate in these forums. | A detailed plan of student-­‐supervisor interactions is included in the individual study plan and discussed with student, supervisors, Departmental Director of Doctoral Education and Admissions Board during the admissions seminar.  Daily, weekly or monthly physical meetings can be complemented by email/Skype/telephone interactions depending on the geographical locations of students and supervisors. | 0 |  |  |  |  |  |  |
| #30  BR  5.5 | It should be ensured that training for all supervisors and potential supervisors is available. | In order to ensure that our supervisors have received adequate training, the School has provided “Supervising Research Degree Students Masterclass” training to 226 Academics that are currently or intending to supervise over the past two years. Training is provided by Dr Margaret Collins, Training for Universities, who is a Specialist Training & Coaching for Researchers and Academics. Supervisors are expected to attend an update training session every three years.  Our PGRs are fully supported by their supervisors who are working in line with Cardiff University regulations to provide an excellent research training environment. In the National PRES 2017 survey 98% of respondents (n= 118) of PGRs agreed that their supervisor(s) had the skills and subject knowledge to support their research. 97% (n= 116) of PGRs had regular contact with their supervisor(s) that they felt was appropriate for their needs. 95% (n= 114) of PGRs agreed that their supervisor(s) provided feedback that helped them to direct their research activities. 91% (n= 109) of PGRs agreed that their supervisor(s) helped them to identify research-associated training and development needs and that these needs were fully supported by the supervisory team (91% agreement). 97% (n=116) were aware of their supervisors' responsibilities towards them as a research student | See <https://www.liverpool.ac.uk/pgr-development/supervisors-and-staff/> There is no easily accessible central record of what training supervisors have taken. This will be remedied in the next few years. All must have taken a course on PhD student supervision during training after first appointment. Recently a requirement for renewal every 5 years (on-line training) has been introduced. A new course about the role of the internal examiner has also been introduced. | The following training elements are available to supervisors:   - **Educational course in supervision of PhD candidates**: [UPED622](http://www.uib.no/emne/UPED622) (NB! Course description currently only in Norwegian, although the course is taught in both Norwegian and English). All new members of the faculty are strongly encouraged to take the course. - **Annual seminars for supervisors** at the Faculty. Since 2010, the Faculty has arranged annual seminars for all PhD supervisors. The seminars acts as forums where supervisors can discuss issues, exchange experiences, and increase their understanding of the activities related to the PhD programme at the Faculty. Although participation in the seminars is voluntary, all supervisors are strongly encouraged to participate. - **e-course for training of supervisors:** The Faculty are in the final stages of establishing an electronic course aimed at supervisors for the PhD candidates at the Faculty. The key elements of the e-course are:  1. General Information (Information about the PhD at UiB, the Faculty of Medicine, and Ethics) 2. Admission (Requirements, training component, supervision) 3. Follow up 4. Examination (requirements, process) 5. Plagiarism and research misconduct   Each element contains questions that the supervisor must answer correctly in order to pass the e-course. | A basic (1-­‐week) course in supervisor training is mandatory for all new supervisors. A web-­‐based course (focus on KI rules) is required for all ‘established’ supervisors as well as for supervisors from other universities.  Advanced courses (1-­‐week) are optional for increasing skills in either pedagogical reflection or in leadership skills. (2017: Principal Investigator course)  A series of optional subject-­‐specific lunchtime seminars are hosted each term for the purpose of ‘refreshing’ or ‘deepening’ of knowledge, and include subjects such as conflict management, research ethics and ILOs. | 2 |  |  |  |  |  |  |
| #31  BR  5.6 | The supervisor-candidate relationship is the key to a successful PhD programme. There should be mutual respect and shared responsibility. | The student-supervisor relationship does not always run smoothly. I developed, trialled and implemented a School strategy (traffic light system) to accelerate the timely resolution of student related issues. The ‘traffic light system’ ensures that appropriate staff are made aware of student/supervisor issues as they arise and that suitable action is taken in accordance with School policy and University regulations. Common issues include; inadequate supervisor/student contact during the period of study, a mismatch in expectations between the supervisor and his/her student and failure of student and/or supervisor to engage (e.g. maintaining contact and/or support) during write-up period. The implementation of a traffic light system has facilitated the timely resolution of student-related issues raised through Progress Monitoring System and in confidential report submitted to the Director of Postgraduate Research Studies by a student or a supervisor. Cases are designated green (expected resolution within 1 month), amber (expected resolution within 3 months) or red light (resolution extended past 3 months). For red light cases, PGR student recruitment by supervisor(s) involved was frozen (irrespective of circumstance). | Matching is undertaken during the admission process. Solving conflict is difficult and there is more than one strategy. In IIB the PGR Pastoral Group may be able to help the student and resolve the situation. The other supervisor(s) is/are important in maintaining contact and progress with the research. Sometimes the secondary supervisor takes on the primary role to resolve conflict. The Head of Department or Institute Director of Postgraduate Research can become involved. If the relationship breaks down permanently, it may be possible for the student to move to a different supervisory team. This may require a major change in the research project. | **Enhance relationship**: The PhD candidate establishes contact with the supervisor prior to the admissions process. Thus, PhD candidates and supervisors are not matched by the Faculty. However, the Faculty aim to enhance the supervisor-candidate relationship in the following ways:   - Regular meetings for PhD candidates at the departmental level - Participation in research school’s network meetings where supervisor and candidate meet regularly   **Solving conflicts:** According to the PhD regulations §6.4, It is the Faculty’s policy that one should always seek to resolve conflicts at the lowest possible level. Cases concerning termination of supervision should always first be discussed at the candidate’s department. If the matter cannot be resolved by the department alone, the case may be transferred to Faculty level. | This is a cornerstone to the KI philosophy, and something that is discussed during the admission seminar with students and supervisors, during the PhD student introduction course, and during supervisor training courses.  Supervisors and students are matched by mutual consent and interaction prior to admission.  Supervisor-­‐student interactions are monitored by the Departmental Director of Doctoral Education, and should any conflict arise the Director has the responsibility to work for a solution of the problem.  Following a completed halftime review, in which the halftime review committee has indicated that they perceive problems in the supervisor-­‐student relationship, then the Departmental Director of Doctoral Education must investigate and work towards a resolution of the problem. | 0 |  |  |  |  |  |  |
| #32  BR  5.7 | Institutional assistance should be provided for career development. This should be continuous, starting from the time of enrolment. | PhD researchers are assisted in career development at Induction. Support by the supervisory team, mentor(s), Director of Postgraduate studies (via the PGR curriculum) continues throughout their candidature. The programme of advice and support is dynamic and reactive in that it is adapted annually in response to student need. This is determined from dashboard responses an annual PGR survey (National and/or School). PhD candidate representatives from each research division work collaboratively with PGR leads from each division and the Director of PGR via the Research Degrees Committee to ensure that student needs are met and that our researchers actively participate and claim ownership for the PGR curriculum.  The PGR Reps also come together as the Postgraduate Research Representatives Forum, which meets at least twice per year and provides a report to the Doctoral Academy Steering Group. | See section BR 4.5. Until 2015 all PhD candidates were required to undertake a career development activity. Since then it has become optional. Activities include placements and internships, seminars offered by IIB and also several annual one-day career events provided by Institutes, Schools, Departments or Doctoral Training Partnerships in the University. Many students attend but without a central record of training it is not possible to give numbers with confidence. | A conscious approach to career development is emphasized from the start of PhD period, mainly through the following ways:   - As a regular element in the mandatory introductory course for PhD candidates ([MEDMET1](http://www.uib.no/en/course/MEDMET1)) the PhD candidates are encouraged to make a career plan. - The Faculty of Medicine offer courses and seminar to PhD supervisors in career guidance. Career guidance are also part of the mandatory supervision training at the Faculty. - All supervisors are expected to include career guidance as a regular element in the general supervision. - A Career Day is arranged for all PhD candidates each year. The Career Day aims to expose the candidates to alternative career paths and to provide them with practical tools on how to find vacancies, write CVs, apply for positions etc. | PhD candidates are informed about career support during the PhD Introduction Course. KI expects supervisors to offer career advice during the PhD. A central career office offices individual support as well as group training (e.g. CV writing, interview training) and also organises annual career fairs at which biotech companies present themselves to early stage researchers. | 1 |  |  |  |  |  |  |
| #33  QD  5.1 | Responsibilities of each supervisor ought to be explicit. | Specific responsibilities of a main supervisor include:   1. giving guidance on: the nature of research in the discipline concerned; the standard of work expected in relation to the qualification-aim; the planning of the research programme, bearing in mind the expected submission deadline; sources, methods and techniques, and specialist research skills required;  the development of professional skills; ethical, intellectual property and/or confidentiality considerations; research integrity and professionalism, and the implications of research misconduct; the drafting of the thesis; 2. ensuring that the student is competent to perform their tasks safely, and that they comply with University and local health and safety procedures; 3. when a student commences their studies at a point other than the start of the academic year, ensuring that they receive the necessary School induction; 4. assessing the student's research and professional skills needs, and encouraging them to participate in training and development opportunities through which they can acquire and practise their skills and apply them to their research work; 5. supporting and monitoring the student's progress through the Research Plan, the planned schedule of meetings and formal contacts, and the reporting requirements of the Research Student Progress Monitoring Procedure; 6. requesting written work from the student and returning it with constructive feedback and within a reasonable time; 7. giving prompt and candid advice about any inadequacy of progress or the standard of the written work being produced, and alerting the director of Postgraduate Research Studies to any difficulties in this regard; 8. being accessible to the student, within reason, and responding to requests for advice and guidance within a reasonable timescale; 9. encouraging students to interact with other researchers, to engage in the research environment, and to take opportunities to present and debate their work, both within and beyond the University; 10. giving guidance on the submission of conference and journal papers; 11. being aware of how to access the appropriate University's Regulations, Codes of Practice and Procedures governing research degrees, and drawing the student’s attention to them as appropriate; 12. ensuring that the student is aware of sources of advice, guidance, support and counselling in the University; 13. alerting the Director of Postgraduate Research Studies to any particular difficulties the student is experiencing which might affect the progress of work; 14. advising the Director of Postgraduate Research Studies of planned absences and suggesting alternative arrangements; 15. reading the draft thesis and advising on its amendment, before formal submission for examination; 16. providing continued guidance to those candidates whose examined work is to be revised and re-submitted for further examination; 17. updating their knowledge and skills with regard to research student supervision;   maintaining a proper and professional relationship with the student. | See <https://www.liverpool.ac.uk/media/livacuk/tqsd/code-of-practice-on-assessment/appendix-2-PGR-CoP.pdf> | The rights and duties of the supervisor are specified in the PhD Regulations § 6, as well as the PhD Agreement (see QD5.4.). | These are clearly defined in the Rules for Doctoral Education:  The principal supervisor shall have the overall responsibility for supervision when it comes to the planning and execution of the research project. The principal supervisor also has, together with the doctoral student, a responsibility to ensure that the doctoral courses and other elements that are specified in the general syllabus and individual study plan are completed. The principal supervisor shall work to ensure that the annual follow-­‐up, as well as the half-­‐time review and defence of thesis, or licentiate degree, are planned and implemented.  The principal supervisor is responsible for ensuring that the funding plan in the individual study plan is drawn up and revised.  A co-­‐supervisor is primarily responsible for providing complementary scientific expertise to the project. The role of the co-­‐supervisor shall be clearly stated in the individual study plan. | 0 |  |  |  |  |  |  |
| #34  QD  5.2 | Supervisors ought to have broad local and international scientific networks. | The supervisor’s publication record and grant capture reassures the School that he/she has suitable networks. | See <https://www.liverpool.ac.uk/media/livacuk/tqsd/code-of-practice-on-assessment/appendix-2-PGR-CoP.pdf> and BR5.3 | The Faculty does not regularly measure if supervisors have suitable networks. However, the networks of the supervisors are evaluated as part of any applications for UiB funding, as well as in the evaluation of the PhD project in the admissions process. | Where possible and as defined by the requirements of an approved individual study plan, the ability of a PhD student to benefit from the local, national and international networks of the supervisor(s) is recommended. Extensive networks might not be expected for more junior, first-­‐time supervisors, and this will not prejudice acceptance of a supervisor. | 0 |  |  |  |  |  |  |
| #35  QD  5.3 | Supervisors ought to assist with career development. | See BR4.5, BR4.7 and BR 5.7 – this point is adequately covered in the above sections. | Candidates say that supervisors assist well with academic career development but for other careers assistance is needed from Institute events, LDC, University careers office. See also BR5.7 | Although the Faculty does not have any specific measures to assist the candidates with career development, all supervisors are encouraged to provide guidance to their candidates regarding this. Both supervisors and to some extent the administration assist with career development on an individual and informal basis. For additional information see BS2.2. | This is an explicit task given to appointed supervisors and instructions to this effect are included in the basic supervisor training course.  The (at least) annual personal evaluation discussion staged by supervisor and student includes discussion of career development (see Personal Evaluation assessment form). | 0 |  |  |  |  |  |  |
| #36  QD  5.4 | Institutions could consider having contracts on the supervision process, signed by supervisor, PhD candidate and head of graduate school. | Each supervisor and student is given a copy of Our Code of Practice at enrolment. The PGR team in the School are required to return a signed & dated copy of the document to certify that they have read the content and will work according to the guidance. | Three month report requires statement signed by candidate and supervisors about how supervision will be conducted. | Yes. According to the PhD regulations § 5.6, admission to the university's PhD programme must be formalized in a written agreement within the framework of the standard agreement for admission adopted by the University Board. The agreement is to be signed by the candidate, supervisor(s), department and the Faculty that the candidate has been admitted to. The agreement governs the parties' rights and obligations during the period of admission and is intended to ensure that the candidate regularly participates in an active research environment, and is to facilitate the completion of the PhD programme within the agreed-upon time. If a supervisor is appointed after the date of admission, this supervisor must sign the agreement immediately after their appointment as supervisor. At least one supervisor must be named at the time of admission, cf. § 5.3. | On enrolment of a PhD student, a dissertation agreement (contract) is set up and signed by the candidate, the supervisor, the co-supervisors, the head of the graduate school and the Dean of Doctoral Studies. | 0 |  |  |  |  |  |  |
| #37  QD  5.5 | The principal supervisor, at least, ought to have some formal training as supervisor. | See above BR5.5 | See BR5.5 | According to the PhD Regulations § 6.1, at least one of the appointed supervisors should have previous experience of supervision of candidates at the master's and/or PhD level. Furthermore, all new members of the scientific staff are strongly encouraged to take the course [UPED622](http://www.uib.no/emne/UPED622) – Educational course in supervision of PhD candidates (cf.BS5.5). In the last few years several members of the scientific staff have successfully completed this course. Furthermore, both new and experienced supervisors are encouraged to participate in the annual supervisor seminar (cf. BS5.5) and the forthcoming e-course for supervisors (cf. BS5.5). | This is a prerequisite for all supervisors, as detailed in BS5.5.  To date 1467 have completed the basic training course and 227 have completed the advanced courses. | 2 |  |  |  |  |  |  |
| #38  QD  5.6 | Supervisors could where possible also act as co-supervisors for PhD candidates at other graduate schools. | Yes | Fairly common since much research involves collaboration with academic staff in other institutes/departments/schools. | Although it isn’t common, on occasions supervisors affiliated with the Faculty of Medicine take on supervision as co-supervisors for candidates at other graduate schools. | We do not have a graduate school system, but supervisors are often involved in the co-­‐supervision of PhD students between different KI Departments, between different KI campuses, and between Swedish Universities. | 0 |  |  |  |  |  |  |
| #39  QD  5.7 | Graduate schools ought to consider appointing a mentor or equivalent for each PhD candidate, in addition to the supervisor team, to discuss programmes from another aspect than the science topic alone. | Yes mentors are appointed in Year 1, mentors change with student needs as prescribed by the candidate.  In order to soothe the transition for postgraduate researchers registered in the School of Medicine, I launched a mentoring scheme that would (i) be richly rewarding for the mentee and mentor and (ii) assist each student to achieve personal and professional growth as he/she progressed and developed within the University and beyond. The scheme, launched in 2013, exemplifies my key contribution as Director of PGR to promoting a high quality teaching and learninenvironment for staff and students. The scheme was identified as an area of good practice at the University’s Annual Review and Enhancement (ARE) exercise in 2014. Feedback from mentees and mentors told me that it provides a developmental platform that nurtures excellent new teachers, promotes the professional training of academic staff, inspires research-led teaching opportunities and underpins the delivery of ‘Research Excellence’ in the School | All candidates have 2 assessors who can act as mentors, and are usually receptive to this role. IIB also has a PGR Pastoral Group of academics and administrative staff so that candidates can (if they wish) select a person distant from their own research area for advice. | The University of Bergen does not have such an arrangement. However, all PhD candidates have two administrative contact persons (one at departmental level and one at Faculty level). In addition, each department have a Head of Research who has an overall responsibility for the follow-up of PhD candidates at the respective department. | Each student, in addition to 2 or more supervisors, has an external mentor as an additional confidant/advisor during the duration of their studies that allows for social monitoring and evaluation of career planning, in addition to that conducted by the supervisor team. | 2 |  |  |  |  |  |  |
|  | | ***6. PhD thesis** | | | | | |  |  |  |  |  |
| #40  BR  6.1 | The PhD thesis should be the basis for evaluating if the PhD candidate has acquired independent research skills and can evaluate work done by others. | This statement is correct for the School of Medicine at Cardiff University. The oral (viva voce) examination is an integral part of the research degree examination process; it is not merely a ritual. An oral (viva voce) examination is mandatory for all PhD  thesis submissions. The Examining Board that is constituted for the purpose of the viva voce examination undertakes the only summative assessment of a Candidate's work. | The thesis and viva voce examination are the basis for evaluating the PhD. | Yes. However, the PhD thesis, trial lecture and public defence (disputation) must all be approved by an independent evaluation committee in order for the candidate to be conferred to the degree Philosophiae Doctor (PhD).  The PhD regulations § 10.2 state that if a written work has been produced in collaboration with other authors, the PhD candidate must follow the norms for co-authorship that are generally accepted in their academic community and in accordance with international standards. In theses that include work with multiple authors, a signed declaration that describes the PhD candidate's input in each work must be enclosed. The statement must be written in the same language as the thesis and must be submitted along with the thesis. The statement is redistributed to the evaluation committee.  For additional information regarding the evaluation of the thesis, please consult the GUIDELINES FOR THE EVALUATION PROCESS OF THE DOCTORAL DEGREE AT THE FACULTY OF MEDICINE, UNIVERSITY OF BERGEN | This is inherent in the general and individual ILOs specified for each PhD student. The annual report for each student includes a self-­‐reflection about perceived progress to this goal, and the halftime review enables a formal assessment of progress towards this end. | 0 |  |  |  |  |  |  |
| #41  BR  6.2 | The benchmark for a PhD thesis in health sciences is the equivalent of three in extenso papers in scientific peer-reviewed international journals. Manuscripts are also acceptable. It is the task of the assessment committee to determine if the material demonstrates 3-4 years of research at international level. | The Convenor of the Examining Board must be satisfied that a prima facie case exists for accepting the submission and referring the thesis for examination. The decision to refer the thesis for examination should not be based on the perceived currency or strength of the research and writing, or whether the submitted thesis meets the criteria for the Award, since only the Examining Board is required to make such an assessment. The Convenor  may, however, wish to take into account the format and presentation of the thesis in relation to Senate Regulations for the Presentation and Submission of Research Degree Theses. If satisfied that a prima facie case exists for accepting the submission and referring the thesis for examination, the Convenor appoints the Internal Examiner, where appropriate, and make(s) the nomination to the Registry of the proposed External Examiner(s).  The thesis should be typed and may be presented for examination in a temporary, but secure binding with a glued spine, which is sufficiently robust to withstand transit to and from the examiners. | University of Liverpool does not require publications, although the majority of students have 1 or more by the time of thesis submission. Timescale of 3 - 4 years means that many students would not have 3 first author papers during this time. The examiners determine whether the thesis demonstrates adequate original research. See <https://www.liverpool.ac.uk/media/livacuk/tqsd/code-of-practice-on-assessment/appendix-7-PGR-CoP.pdf> and <https://www.liverpool.ac.uk/media/livacuk/tqsd/code-of-practice-on-assessment/appendix-8-PGR-CoP.pdf> | As stated in Guidelines for publications in doctoral dissertations at the Faculty of Medicine, point III, a doctoral dissertation should normally consist of three scientific articles prepared for international journals with a peer review system.  As a general rule, the doctoral candidate should be the principal author of at least two articles. Where the candidate is not the principal author, he / she should have contributed considerably in the collection of data, interpreting of results and writing of the article.  The number of articles will depend upon the extent and quality of each article, and on the candidate’s contribution. If the candidate has put an unusually large amount of work into one article, and that article is of a very high standard, the number of articles may be reduced. Under these conditions, one and the same article may be included in several doctoral theses. Manuscripts are also acceptable. However, the Faculty recommends manuscripts that are either published or accepted for publication.  It is the task of the evaluation committee to determine if the material demonstrates 3 years fulltime of research at international level.  For additional information reference is made to supplementary the PhD regulations, Guidelines for publications in doctoral dissertations at the Faculty of Medicine and Guidelines for the evaluation process of the doctoral degree | Most theses from KI are compilation theses, based on a number of separate original papers (not reviews). One of the constituent papers can be a systematic survey article, for example a meta-­‐analysis. The requirement for such an article is that is creates new knowledge.  At least half of the constituent papers that are included in the compilation thesis must have been accepted for publication in a peer-­‐reviewed journal. (2017: 2/3 papers published)  The number of constituent papers in a compilation thesis varies, but they must have a scope and quality that in total, in the opinion of the Examination Board, is equivalent to four years of full-­‐time doctoral education. The doctoral student's contribution to the constituent papers must be clearly identified. | 2 |  |  |  |  |  |  |
| #42  BR  6.3 | In defining the benchmark for a PhD thesis, the assessment committee should take account of the provisos listed in the Annotations, for example the annotation indicating that fewer than three papers may be accepted if published in highly rated journals. | A candidate is at liberty to publish the whole or part of the work produced during his/her candidature prior to its submission as a whole, or part of a thesis, provided that in the published work it is nowhere stated that it is in consideration for a higher degree.  Research work already published, or submitted for publication, may be incorporated in a thesis. With the exception of Doctoral Degrees by Examination and Thesis, papers themselves may not be included in the body of a thesis but may be adapted to form an integral part of a thesis and make a relevant contribution to a main theme. Publications derived from the work described in a thesis may be appended as supplementary material, not to be assessed, at the back of a thesis. Where portions of investigation were conducted in collaboration with, or with the assistance of, others, the extent and nature of these contributions should be highlighted clearly in acknowledgements, as well as referenced within the main body of the text. Candidates must ensure that material authored by a third-party that has been used in their thesis has been sufficiently acknowledged.  The criteria for the award of the degree of PhD (by Published Works) are the same as those established for the degree of PhD, | See BR6.2 | A candidate is at liberty to publish the whole or part of the work produced during his/her candidature prior to its submission as a whole, or part of a thesis, provided that in the published work it is nowhere stated that it is in consideration for a higher degree.  Research work already published, or submitted for publication, may be incorporated in a thesis.  (As for Cardiff)  See also BR6.2. | The pre-assessment by the examination committee before the formal thesis defence is approved centres on conforming that the work presented is what should be expected for a 4-year time equivalent period of research training. The number and quality of the component research papers/manuscripts is thus considered at this time, and provision for fewer, higher impact publications can therefore be accepted at this stage. | ? |  |  |  |  |  |  |
| #43  BR  6.4 | In addition to papers, the thesis should include a full literature review and full account of aims, method, results, discussion and conclusion. | The PhD (by Published Works) submission comprises: a list of the works submitted; the published works to be examined; a critical commentary, any other documentation which may be required. Candidates shall be required to provide a critical commentary of some 5,000-10,000 words giving an evaluation of the field in which they have worked, and indicating also the original contribution to learning in that field which in their opinion their work has made. Candidates may submit work(s) done in collaboration with others in support of the candidature, but such work shall be accompanied by a statement signed by each collaborator indicating the nature and amount of the work  done by the candidate. | See <https://www.liverpool.ac.uk/media/livacuk/tqsd/code-of-practice-on-assessment/appendix-7-PGR-CoP.pdf> section 3 for contents of thesis and note 3e for statement on presentation of thesis if made from papers. Candidate must indicate her/his contribution to each paper. | As stated in Guidelines for publications in doctoral dissertations at the Faculty of Medicine, point III, in addition to the individual articles, the thesis should include a general presentation of the scientific results with a detailed, up-to-date comparative discussion. It should demonstrate scientific overview and maturity, as well as the ability to penetrate scientific problems. Normally, the theses include a full literature review and full account of aims, method, results, discussion and conclusion. In addition, methodological considerations are also normally emphasised in the theses. In the methodological considerations the candidate evaluates the choice of methods in the study, and discusses the strengths and weaknesses of the chosen methods. Such considerations are not necessarily clearly identified in each individual article.  For additional information reference is made to supplementary the PhD regulations, Guidelines for publications in doctoral dissertations at the Faculty of Medicine and Guidelines for the evaluation process of the doctoral degree | A KI thesis usually comprises of 3 sections:  An overview of the subject area, with the purpose of demonstrating both broad and specific knowledge of the subject area, with appropriate referencing.  A summary of the work conducted, including *Aims, Methods, Results* and *general Discussion* and *Conclusions.* This should not be copied from the constituent papers/manuscripts, but instead provide the student with the opportunity to contextualise their findings with respect to the presented literature review, and to reflect on their learning experiences.  The published papers and manuscripts. See also:  https://internwebben.ki.se/sites/default/files/riktlinjer_ramberattelse_eng_2012.pdf | 0 |  |  |  |  |  |  |
| #44  BR  6.5 | If the thesis is presented in other formats (e.g. as single monograph), the assessment committee should ensure equivalence to the above benchmark. | University considers that a series of academic papers without being adapted, in lieu of a conventional format, is not generally acceptable for submission as a research degree thesis. The reasons for this include:  1. a research degree thesis should present a cohesive narrative, that is stylistically coherent and avoids repetition;  2. a thesis should include sufficiently detailed descriptions and evaluations of relevant methodologies, research protocols, theoretical approaches, method development, experiments, etc., to allow the examiners to assess the work against the criteria for the award;  3. many academic papers are multi-authored, and the candidate's individual contribution is not readily apparent. | The majority of theses are presented as monographs. | Almost all PhD theses are presented as collection of articles.  other formats (monograph). In a five year perspective (2010/2015), 0.85 % (four out of 472 theses) was presented in other formats (monograph).  As stated in Guidelines for publications in doctoral dissertations at the Faculty of Medicine, point II Unpublished monographs will be evaluated according to the same criteria as a collection of printed articles with their summary. It is to be understood that the minimum requirements regarding scientific quality and quantity are identical.In a one year perspective (2014/2015), 1.2 % (one out of 83 theses) was presented in | A doctoral thesis may also be presented in the form of a monograph thesis. Special rules of review then apply | (0) |  |  |  |  |  |  |
| #45  BR  6.6 | A PhD thesis in clinical medicine should meet the same standards as other PhD theses . | Yes | Yes. (The MD degree has different requirements; see Ordinance 58A <https://www.liverpool.ac.uk/corporate-governance-and-support-office/how-we-are-governed/programme-ordinances/>) | Yes. The Faculty only offer one PhD programme, and doesn’t distinguish between PhD theses in clinical medicine and other PhD theses in other academic areas. | There is no distinction between clinical, preclinical or other student PhD thesis requirements – the same rules apply throughout. | 0 |  |  |  |  |  |  |
| #46  QD  6.1 | The thesis ought to be written and optimally also defended in English, unless national regulations stipulate otherwise. An abstract of the thesis should be published in English. | All theses shall be presented in English or Welsh. | All in English (except for Departments working in other languages, e.g. Department of French) | According to the PhD regulations § 10.4. The thesis must be written in English, Norwegian, Swedish or Danish. If the candidate wishes to use a language other than these, an application to this effect must have been submitted and approved at the time of admission, (cf. § 5.1.). Still, the Faculty advises all candidates to write their thesis in English. This is also the case for Norwegian-speaking candidates. | The thesis is recommended to be written in English. Translation of the scientific *Abstract* to Swedish is an option that some Swedish students adopt.  The thesis defence is almost always defended using English, and this is the KI recommendation. In the case of an ‘all-­‐Swedish faculty opponent/ committee’ and at the expressed wish of either opponent or PhD student, the Swedish language is sometimes (rarely) used (most typically by some clinical students). | 0 |  |  |  |  |  |  |
| #47  QD  6.2 | If articles/manuscripts are joint publications, co-author statements ought to document that the PhD candidate has made a substantial and independent contribution. Ownership of results from PhD studies ought to be clearly stated. | Where portions of investigation were conducted in collaboration with, or with the assistance of, others, the extent and nature of these contributions should be highlighted clearly in acknowledgements, as well as referenced within the main body of the text. Candidates must ensure that material authored by a third-party that has been used in their thesis has been sufficiently acknowledged. | If thesis contains publications, candidate must explicitly state their contribution. This can be discussed in the viva examination. University policies do not allow a piece of research to be submitted for more than one award. Software to detect copying (e.g. Turnitin) can be used on a thesis if there is concern. | According to the PhD regulations § 10.2, joint work is accepted for assessment (including as one of several works, cf. § 10.1), provided the candidate's contribution represents an independent effort that can be identified to the extent necessary for the assessment. In such cases, statements must be obtained from the other authors, or their central representatives, and possibly from others involved in the work, in order to identify the contribution made by the doctoral candidate. Joint work should, as far as possible, be agreed upon in advance.  If a written work has been produced in collaboration with other authors, the candidate must follow the norms for co-authorship that are generally accepted in their academic community and in accordance with international standards. In theses that include work with multiple authors, a signed declaration that describes the candidate's input in each work must be enclosed.  Furthermore, the GUIDANCE ON REQUIREMENTS RELATING TO DOCTORAL DISSERTATIONS FOR  THE PhD DEGREE AT THE FACULTY OF MEDICINE, UNIVERSITY OF BERGEN states that under exceptional circumstances, a complete thesis may be the joint effort of two doctoral candidates. In such cases, the thesis should be equivalent in scope to two normal doctoral theses. In addition, the independent efforts of each individual candidate must be clearly defined and should be equivalent to about half of the scientific work involved. This also applies to the summary. | For each published article, submitted article or manuscript included in the PhD thesis, the contributions of the defending PhD student are explained as part of the PhD thesis defence application. In this paperwork it is clearly stated to what degree the defending student has contributed to the scientific concept, conduct, interpretation and publication of each article. It is also clearly indicated if the articles will be used in another PhD thesis. In accordance with Swedish law a PhD student owns all of their own results, so this is not an issue. | 0 |  |  |  |  |  |  |
| #48  QD  6.3 | PhD theses ought to be published on the graduate school’s homepage, preferably in extenso. If patent or copyright legislation prevent this, at least abstracts of the theses ought to be publicly accessible. | Once the Examining Board has made recommendation for an award, and has approved the required corrections where applicable, the candidate will be required to upload the final version of the thesis into the University's digital repository before confirmation of the award. | All theses are published from University repository, with an embargo period if necessary. See <https://www.liverpool.ac.uk/student-administration/research-students/submission-and-examination/final-submission/> | According to the PhD regulations § 12.7, the thesis must at the latest be publicly availa be made available in the form ble two weeks before the public defence is held. The thesis must in which it was submitted for assessment, alternatively as revised on the basis of the committee's preliminary remarks, cf. § 11.5.  Furthermore, according to the PhD regulations § 12.8, the candidate must prepare a press release well in advance of the disputation, and submit it to the Faculty for approval. The press release must be prepared in accordance with the adopted template. The Faculty is responsible for sending the press release to the Division of Communication no later than two weeks before the public defence.  The time and place of the public defence must is announced to the public ten (10) working days in advance.  All PhD candidates are also invited to publish their thesis in Bergen Open Research Archive (BORA). | An electronic PDF copy is made of each doctoral thesis and published at a defined KI thesis database website ([http://publications.ki.se/xmlui/handle/10616/1?locale-­‐](http://publications.ki.se/xmlui/handle/10616/1?locale-)attribute=en) The electronic version is not uploaded until the thesis defence has been approved by the KI Dissertation Committee. | 0 |  |  |  |  |  |  |
| #49  QD  6.4 | There could be a lay summary of the thesis in the local language. | All theses shall be presented in English or Welsh. Quotations and technical terms, however, may be given in the language in which they were written. There is no prescribed format for the summary only that it be 300 words or less | There is not a lay summary. | A lay summary of the thesis is in the format of a press release (cf. PhD regulations 12.8). | While this is common for Swedish PhD students it is not a requirement, as English is the official language of Doctoral Education at KI. | 2 |  |  |  |  |  |  |
|  | | ***7. Thesis assessment** | | | | | |  |  |  |  |  |
| #50  BR  7.1 | Acceptance of a PhD thesis should include acceptance of both written thesis and a subsequent oral defence. | See BR6.2 for conditions relating to acceptance of thesis. The oral examination shall be arranged and conducted in accordance with  the University's 'Procedures for the Arrangement and Conduct of Research Degree Examinations'. Examining Boards, in recommending a candidate for the degree, shall certify that the thesis submitted by the candidate substantially covers the programme of research approved for him/her. The viva voce examination may serve different purposes, according to a  Candidate's differing qualities and those of the written thesis, but the essential purposes of the viva voce examination are:  - to enable the Examiners to assure themselves that the thesis is the Candidate's own work;  - to enable the Examiners to assure themselves that the Candidate understands the research that s/he carried out and what s/he has written in the thesis;  - to enable the Examiners to assess the Candidate's ability to locate his/her work within the broader context of the particular field of scholarship to which the project relates;  - to give the Candidate an opportunity to defend the thesis and to clarify any obscurities or weaknesses in it: this is particularly important in borderline cases;  - to enable the Examiners to explore how the thesis might be raised to the required standard should they be unable to recommend the award at this stage.  Examiners may also take the opportunity to discuss with the Candidate their subsequent research or professional direction and/or to explore prospects for publication. | Candidate has to submit 2 soft-bound printed copies of thesis and a pdf. Printed copies of the thesis are sent to Internal and External Examiner. They have to provide a written Initial Report, indicating the probable award, before the viva. Within 3 months of thesis submission the candidate and examiners meet at University of Liverpool for viva voce examination (so only 3 people present normally). Length is variable but normally 2-3 hours. The candidate is told the outcome at the end of the viva. Examiners have to provide a written joint report after the viva, including stating the award, any corrections required and who supervises the corrections. For details see <https://www.liverpool.ac.uk/media/livacuk/tqsd/code-of-practice-on-assessment/appendix-8-PGR-CoP.pdf> | The evaluation procedures consists of three stages:  **Evaluation of the thesis**: The evaluation committee is requested to state whether or not the thesis satisfies the formal and real requirements set out in the PhD Regulations. The Guidelines regarding requirements for a PhD thesis at the Faculty of Medicine, University of Bergen should be used as a support for the committee’s evaluation. The evaluation committee’s decision is stated in the evaluation report. The statement of the committee (evaluation report) should first and foremost present a clear and unambiguous conclusion as to whether the qualitative and quantitative scientific requirements have been met, so that the thesis can be defended for the degree of PhD. The statement should provide a well-grounded justification for this conclusion.  **Trial lecture over a given topic**: The chairman of the evaluation committee is responsible for ensuring that the title of the trial lecture on a topic of the committee’s choice is received by the Faculty at least one month before the planned trial lecture. The title of the lecture should be submitted in writing. The title will be treated as confidential until it is given to the doctoral candidate, 10 working days before the date of the trial lecture. The trial lecture will be given in a central auditorium and at a time which permits it to be included in the Faculty’s basic and advanced teaching schedule. The subject of the lecture should be taken from central areas of clinical, paraclinical or preclinical medicine, and should be of interest to both students and staff at the university and hospital. The length of the lecture will be 45 minutes, followed by questions and discussion. The trial lecture must be approved by a committee appointed by the Faculty before the public defence takes place.  **Public defence:** The doctoral candidate initiates the defence by presenting the objectives of the scientific study and the results it has obtained. The introduction should not exceed 30 minutes. Thereafter, the defence continues in the form of a discussion of the thesis involving the opponents and the doctoral candidate. At the public defence the first and second opponents each submit an oral opposition, the point of which is to present a critical analysis of the thesis. Central aspects of the thesis are discussed with the doctoral candidate, on this occasion in greater detail than in the written statement.    For additional information, reference is made to the PhD regulations § 12 and § 13, and the GUIDELINES FOR THE EVALUATION PROCESS OF THE DOCTORAL DEGREE AT THE FACULTY OF MEDICINE, UNIVERSITY OF BERGEN | Before the thesis is submitted for printing, the Examination Board shall conduct a preliminary review in which the thesis' constituent papers are reviewed, together with details of the individual contribution of the PhD student, record of their formal learning experiences and their perceived performance as summarised by the main supervisor.  If accepted, the student defends his/her thesis before a faculty opponent and an Examination Board comprising of 3 senior researchers  A public thesis defence typically has the following format:  A brief summary of the subject area is given by the faculty opponent (10-­‐20 min).  The student gives a short research seminar to present their work (20-­‐30 min).  The opponent and PhD student discuss the thesis work (1-­‐2 hours).  The examination board and PhD student discuss the thesis work (30 min).  Questions from the audience are then permitted. | 0 |  |  |  |  |  |  |
| #51  BR  7.2 | PhD degrees should be awarded by the institution on the recommendation of the assessment committee which has evaluated the thesis and the oral defence. | Yes | Yes | Yes. According to the PhD regulations, § 14, The University Board confers the philosophiae doctor degree on the PhD candidate on the basis of the report that the trial lecture and defence have been approved.  The diploma is issued by the institution. The diploma states the title of the thesis for which the PhD degree was awarded. Information about the academic training programme the PhD candidate has participated in is enclosed as a Diploma Supplement. | The Examination Board shall assess the thesis and its public defence with regard to:  the respondent's presentation of the thesis at the public defence, including the discussion with the opponent, and the ability to answer questions and discuss the significance of the results within the field of research  the quality of the comprehensive summary  the scientific content of the constituent papers  the fulfilment of the learning objectives of the doctoral degree.  Against the background of this assessment, the Examination Board comes to a decision to award a grade of either pass or fail. | 0 |  |  |  |  |  |  |
| #52  BR  7.3 | The assessment committee should consist of established and active scientists without connection to the milieu where the PhD was performed and without conflict of interest. Min. two should be from another institution. | The assessment committee has 3 members, a chair, internal examiner and external examiner (two external examiner for a staff candidate). Typically the chair is from within the institution but not always. The assessment panel is nominated by the student’s main supervisor. Approval is required from the School and University level who conduct checks to ensure that the examiners are eligible and that there is no conflict of interest (there is a specific question on the nomination form). | The assessment committee in Liverpool (as is typical of UK) is two people. The External Examiner is a specialist in the field of candidate’s research, often eminent, not connected with the candidate and not employed by University within at least 5 years. The Internal Examiner has knowledge of the candidate (usually was an Assessor, and is aware of likely Internal Examiner role from the start of PhD). Examiners are appointed via a form. Name(s) of proposed External Examiner is put forward by supervisor at least 2 months before thesis submission, with short case and biography. Proposal is reviewed (delegated to Institute Director of Postgraduate Research) and then by Faculty Director of Postgraduate Research. If approved, administrators contact Examiners and send thesis. See details in:  <https://www.liverpool.ac.uk/media/livacuk/tqsd/code-of-practice-on-assessment/appendix-8-PGR-CoP.pdf> | The PhD regulation’s, § 11.4, state that the Faculty is to appoint an expert assessment committee consisting of at least three members to assess the thesis and its defense. The department in question proposes committee members. The proposal should show how the committee as a whole covers the field(s) addressed in the thesis. The Faculty assesses the composition of the evaluation committee and ensures that all members are competent and impartial. The provisions on impartiality in § 6 and following of the Public Administration Act apply to the members of the committee; see also § 10 of the Act. If approved, the Faculty appoints the proposed evaluation committee. The Faculty will then also appoint one of its representatives as chair of the committee. To the extent possible, two of the committee members should have no affiliation with UiB. At least one member should be from a foreign institution of higher education. The members must hold doctorates or equivalent academic competence. The gender of the PhD candidate should be represented on the committee. The committee shall normally be composed in such a manner that both genders are represented. | All of the members of the Examination Board must be associate professors or professors.  All of the members must be experts in the field of the project. The combined expertise of the Examination Board shall cover the entire content of the thesis.  The members of the Examination Board must be independent of and unbiased in relation to the doctoral student, the supervisors and the project.  If possible, at least one member of the Examination Board shall have been a member of the board at the doctoral student’s half-­‐time review.  Only one of the members may belong to the same department as the doctoral student (or the principal supervisor, in cases when the principal supervisor is in a different department as the doctoral student).  At least one member shall come from another university. | (0) |  |  |  |  |  |  |
| #53  BR  7.4 | The supervisor should not be a member of the assessment committee. If local regulations require this, the supervisor should not have a vote. | No member of the Candidate's supervisory team can be appointed as a member of the examination board.  A Candidate's supervisor cannot assume the role of Convenor or Chair. Where the Head of School is the supervisor s/he must appoint other staff member(s) to these roles. No member of the Candidate’s supervisory team can act as the Internal  Examiner. A supervisor may, however, be invited to attend the viva voce examination. | Yes. The supervisor is not allowed to be present during the viva and is not supposed to be involved in making arrangements for the viva. | Yes. According to the PhD regulation’s, § 11.4, the appointed supervisor may not be a member of the committee, but may if necessary be called for discussions in the committee in order to account for the supervision and the work on the thesis. The appointed supervisor also may not be the administrator of the committee's work or chair the public defence. | None of the supervisors take part in any of the formal assessment of the PhD. From 2013 it is recommended that the main supervisor *not* act as chairperson during the thesis defence, as has been the previous KI tradition. | 0 |  |  |  |  |  |  |
| #54  BR  7.5 | If the assessment of the thesis/defence is negative, the PhD candidate should normally be given an opportunity to rewrite/an additional defence. | If the assessment panel does not approve the award but recommends that the candidate be allowed to re-submit on one further occasion, the candidate is given 12 months in which to do so. They are provided with a list of thesis deficiencies by the assessment panel. It is the responsibility of the supervisor to support the candidate through the resubmission period. | Resubmission within one year is possible if examiners consider thesis is not adequate for PhD. Examiners must provide detailed information on modifications required. In addition, the degree of Master of Philosophy can be offered if examiners consider revision/further viva will not reach PhD standard. | According to the PhD regulation’s, § 12.5, a thesis which was not approved at a previous assessment may be submitted for reassessment in a revised form, either as the only work or as one of several related works. A thesis which is not approved for public defence may be submitted in a revised form no earlier than six (6) months after the institution made this decision. A new assessment can only be made once.  In the event of resubmission, the PhD candidate must clearly state that the work has been assessed previously and not found worthy of a public defence.  A thesis which has been revised should be submitted no later than one year after the candidate is informed of the decision made by the Faculty Board. | The Examination Board must provide a written justification when awarding a fail. The doctoral student will then have the opportunity to, at a later date, re-­‐apply for a public defence of their thesis. However, there is no obligation on the part of supervisors, the department or KI, to cover the additional costs that result from a renewed defence of thesis, or to support the doctoral student after a failed doctoral examination beyond the expiry of the existing appointment. | 0 |  |  |  |  |  |  |
| #55  BR  7.6 | The oral examination should be detailed enough to ensure that the thesis is the candidate’s own work, that the intended training goals have been achieved, and that the candidate is able to put the results into scientific context. | See BR7.1  The viva voce is an oral defence of the thesis NOT a formality.  The PhD candidate does not give a formal lecture | There is no set format for the oral defence. It frequently starts by asking the candidate to give an overview/summary of the project and then goes through the thesis chapter by chapter. Questions are mainly from the External Examiner and continue until both are satisfied.  The viva is not a traditional formality. The PhD candidate does not give a lecture in IIB; in some other Institutes at Liverpool candidates typically give a seminar prior to the viva. | Prior to the oral defence of the thesis, the PhD candidate is required to give a formal trial lecture. The topic for the trial lecture is provided by the evaluation committee and given to the candidate 10 working days before the date of the trial lecture. The topics should be related to, but not identical to the topic of the thesis. The purpose of the trial lecture is to ensure that the candidate possesses good knowledge about related research fields, and is able to consider his/her own work in a broader scientific context.  The doctoral candidate initiates the defence by presenting the objectives and results of the scientific study. The introduction should not exceed 30 minutes. The defence then continues in the form of a discussion of the thesis involving the opponents and the doctoral candidate. The first opponent in a normal defence has 60-120 minutes at his/her disposal, and the second opponent has 45-60 minutes. Opposition ex auditorio is permitted. Such contributions should be well prepared and precise, and should aim to enrich the scientific discussion. Anyone present may participate. The chairman of the evaluation committee may also present his/her opposition in this way. Opposition ex auditorio takes place before the second opponent takes the floor.  For more information, please see [the Faculty’s guidelines for the evaluation process for the doctoral degree.](file:///P:\7.0%20Forskningsadministrasjon\09.%20Forskerutdanning\Reglement%20og%20veiledere\Engelsk\Guidelines_for_the_evaluation_process_for_the_doctoral_degree%20Vedtatt%2005.09.16.pdf) | The formal thesis defence includes a research seminar summarising the work conducted by the candidate. Thereafter the faculty opponent an subsequently the 3 examination committee members ask questions to assess the candidate’s attainment of learning outcomes. It is inherent from this question-answer-discussion session whether the candidate has been sufficiently trained and has conducted the research themselves. | 2 |  |  |  |  |  |  |
| #56  QD  7.1 | The oral defence ought to be open to the public. | Student, Chair of the Examination Board, External Examiner and Internal Examiner | It is not open to the public. Only the candidate and the 2 examiners can attend (plus an independent chair if, for example, the candidate is a member of University staff or the thesis is a re-submission) | According to the PhD regulation’s § 13.2, the defence is open to the public. | The PhD defence is open to the public. Only the subsequent deliberation of the 3 PhD members of the thesis committee and the faculty opponent are closed proceedings, during which the final decision of approval/non-­‐ approval is made. | 2 |  |  |  |  |  |  |
| #57  QD  7.2 | Where possible at least one member of the assessment committee could be from another country. | Few. | Few. | According to the PhD regulation’s § 11.4, to the extent possible, two of the committee members should have no affiliation with UiB. At least one member should be from a foreign institution of higher education. | A PhD defence comprises a detailed examination/discussion by an appointed faculty opponent, and further examination/discussion by an appointed panel of 3 experts within the scientific field. At least one member of the panel must arise externally from KI. It is more common in Sweden that an international guest assumes the role of faculty opponent. | 2 |  |  |  |  |  |  |
| #58  QD  7.3 | Apart from the thesis, the institution ought to ensure that sufficient transferable skills have been acquired during the PhD programme. | Yes – already covered above | All students acquire some transferable skills during the PhD programme but there is no accessible formal record or assessment. | The Faculty ensures that our PhD candidates acquire transferable skills during the doctoral training:  **Communication skills**: As an obligatory part of the training component, all PhD candidates must complete dissemination activities equivalent of 6 ECTS. Such dissemination activities include the following:   - participation in national, regional or international conferences with either poster of oral presentation - Original academic or popular-scientific lecture - Popular science article/feature article within the candidate’s academic discipline   In addition to obligatory activities, all candidates are encouraged to convey their research consecutively during their doctoral education. In such ways, candidates are considered to acquire extensive communication skills.  **Teamwork:** During the doctoral education PhD candidates will gain much experience with teamwork, for instance by working in research teams and take part in research groups.  **Project management:** Perhaps one of the most important transferable skill candidates acquire during their doctoral education is to manage their PhD project from the initial to final stages. Thus project management is an important transferable skill that PhD candidates acquire during their doctoral education | The inclusion of transferrable skill training and evaluation of progression towards this end are inherent in the PhD programme described in the individual study plan, which is approved on admission and evaluated thereafter by the Department. | 2 |  |  |  |  |  |  |
| #59  QD  7.4 | The competences developed during the PhD programme could be documented in a portfolio. This documentation could be evaluated by the assessment committee and form part of their decision concerning the award of the PhD degree. | Yes – already covered above | There is an on-line facility for PhD candidates to record a portfolio. However, it is not assessed as part of the decision to award Ph.D. | The Faculty does not provide a certain portfolio of developed competences. However, the PhD candidate has to present a public lecture on a theme before acceptance for defending the thesis. The theme is given by the PhD evaluation committee. Such a lecture is taken as a proof obtained both scientific and transferable skills. In addition to the document of an approved thesis there is a diploma supplement listing the course portfolio for the candidate. | An electronic PhD portfolio including the learning outcomes defined at admission and an annual review of progress towards their attainment will be introduced in 2019. This document will become part of the thesis defence application. | (0) |  |  |  |  |  |  |
|  | | ***8. Structure of Graduate School** | | | | | |  |  |  |  |  |
| #60  BR  8.1 | The graduate school should have sufficient resources for proper conduct of PhD programmes. This includes resources to: Support admission of PhD candidates, implement the PhD programmes of the PhD candidates enrolled, assess PhD theses and award PhD degrees. | So that students can undertake research effectively, the School provides the most appropriate working accommodation, equipment and resources that it can. The facilities made available will vary depending on the particular needs of the academic discipline and on the student's mode of study. The School has appropriate administrative arrangements in place to address the following,  .1 recruitment and admission of research students;  .2 School induction programmes;  .3 training and development requirements;  .4 supervision arrangements, including mechanisms by which these may be changed;  .5 monitoring academic progress, requirements for progression and procedures for unsatisfactory progress, and examination arrangements;  .6 student consultation and feedback;  .7 student entitlements, including access to funding for conferences, research costs, travel and training, and teaching opportunities.  The above are clearly described in the School PGR Handbook, guidance notes and web information.  The Head of School has an appointed Director of Postgraduate Research Studies, who is experienced in the supervision of research students, and to whom responsibility for managing postgraduate research activity on a routine basis is delegated. The Head of School monitors the effectiveness of the Director of Postgraduate Research Studies through the appraisal process | LDC currently has very few resources of people or money (currently, Director, Manager and a few administrators; is not a budget-holder) but is being developed into a larger structure. Existing administrative and PGR development staff are being transferred into LDC.  The financial structure at University of Liverpool has devolved budgets to Institutes (in HLS). Thus the Institutes support admission of PhD candidates and implements the PhD programme. Institutes also assess the PhD theses (with oversight from Faculty Director of Postgraduate Research and University administration that is likely to be incorporated into LDC). The University awards the PhD degree (organises the administration and graduation ceremony) | **Administrative resources**: PhD are supported and followed up consecutively by the research administration at the Faculty and the departments. Each candidate has at least one administrative contact person at their respective department and one administrative contact person at the Faculty. The research administrations supports the candidates in all phases of the PhD programme, from admission to the award of PhD degrees, and ensures that all conditions are in place in order for candidates can complete their PhD degree successfully within the estimated time period.  **The Programme Board**: The Programme Board is responsible for ensuring scientific coordination and quality assurance in the PhD programme at the Faculty.  **Organised feedback and regular reporting:** Through the application process, the candidates receive feedback from the research environment regarding the design and planned implementation of their proposed PhD project. They also receive feedback from the research environment on their preliminary work and progress in the PhD programme through completion of the midway evaluation. In addition,  through regular reporting, both candidates and their supervisors are encouraged to give feedback about the status and progress of the PhD project (see point BS4.8).  **Assessment of PhD theses and award of PhD degree**: The Faculty has well established routines for the final stages in the PhD programme, and candidates are offered both administrative and scientific support in these stages (see BS7.1. for an overview of the evaluation phase). The University Board confers the philosophiae doctor degree on the PhD candidate on the basis of the report that the trial lecture and disputation have been approved. | The KI Board of Doctoral Education has overall responsibility for doctoral education within KI, and this is delegated to each Departmental Head.  The Board provides departmental funding for appointment of both Study Administrator and Director of Doctoral Education.  The Board also economically supports a Central Director of Doctoral Education, as well as several administrative officers responsible for the formal administration of doctoral education within KI, and finances the thematic Doctoral Education Programmes that are responsible for running diverse educational activities including all formal teaching courses required for PhD students.  In addition, the Board has responsibility for continual development of Doctoral Education with respect to formal practices, quality assurance and follow-­‐up. | 0 |  |  |  |  |  |  |
| #61  BR  8.2 | The graduate school should have a website in English and possibly also the national language including transparent information about the content of PhD programmes and the policies of the graduate school. | The Medical School’s website provides an overview of the programmes available, their structure, the research fields that can be studied and career prospects. The website also provides information on the entry requirements, the fees and funding and how to apply.  <http://www.cardiff.ac.uk/study/postgraduate/research/programmes/programme/medicine> | Organisation at Liverpool means current arrangements are as follows:  University website has section to search for postgrad research degrees (<https://www.liverpool.ac.uk/study/postgraduate-research/> ) and LDC site (<https://www.liverpool.ac.uk/study/postgraduate-research/liverpool-doctoral-college/> ). Separate webpages provide policies on postgraduate degree (<https://www.liverpool.ac.uk/student-administration/research-students/> ). Individual institutes have more information on their own PhD programmes (e.g. <https://www.liverpool.ac.uk/integrative-biology/postgraduate-study/> ). Funded Doctoral Training Partnerships also have their own websites (e.g. <https://acce.shef.ac.uk/> ) explaining their programmes and policies. | Information about [the Doctoral Education at the Faculty of Medicine](http://www.uib.no/en/mofa/64913/doctoral-education-faculty-medicine-and-dentistry) at the University of Bergen is available on our websites. The website also provides information about the [Faculty Management](http://www.uib.no/en/mofa/66718/faculty-management), our [research schools](http://www.uib.no/en/mofa/63870/research-schools) and [core facilities](http://www.uib.no/en/mofa/64790/core-facilities). Both general information about [the PhD degree at the UiB](http://www.uib.no/en/phd/74305/phd-degree) as well as more specific information about the content of [the PhD programme at the Faculty of Medicine](http://www.uib.no/en/mofa/63903/different-stages-phd-period), is available on our websites. Furthermore, [regulations and guidelines](http://www.uib.no/en/mofa/65665/regulations-and-guidelines) regarding the PhD programme are also published on our websites, including the PhD regulation, guidelines about the methods used for assessing PhD candidates and the requirements for the PhD thesis. The formal framework for following the progress of individual PhD candidates is described in our website regarding [the annual progress report](http://www.uib.no/en/mofa/81634/annual-progress-report) and the midway evaluation. In addition, each department provide information about their own midway evaluation:  [Midway evaluation at the Department of Biomedicine](http://www.uib.no/en/course/MEIBM900)  [Midway evaluation at the Department of Clinical Medicine](http://www.uib.no/en/course/MEK1900)  [Midway evaluation at the Department of Clinical Science](http://www.uib.no/en/course/MEK2900)  [Midway evaluation at the Department of Clinical Dentistry](http://www.uib.no/en/course/MEIKO900)  [Midway evaluation at the Department of Global Public Health and Primary Health Care](http://www.uib.no/en/course/MEIGS900)  [The Programme Board](http://www.uib.no/en/mofa/93296/programme-board-phd-programme-pfu-faculty-medicine-and-dentistry) ensures quality assurance and regular review to achieve quality improvement in the PhD programme.  All websites above are available in both Norwegian and English unless otherwise specified. | Web address for the graduate school:  http://www.medunigraz.at/en/phd-medizin/  Web address of the Office for Doctoral Studies: http://www.medunigraz.at/themen-studieren/phd-medizin/office-for-doctoral-studies/ | 0 |  |  |  |  |  |  |
| #62  BR  8.3 | Merit should be given for courses taken elsewhere or other relevant experience. | Merit is given for Maters degree attainment and for previous research and professionally accredited courses at the admissions stage of a PhD candidature. | Courses and previous research provide training that helps with the doctoral research programmes but there is no explicit credit/merit. All research presented in thesis must be undertaken while candidate is registered for PhD. PhD is awarded for the thesis (and viva) alone. | According to the Guidelines for calculating course credits in the PhD programme, a total of 30 ECTS must be approved as the training component in the doctoral training. Research courses can be organised by the Faculty of Medicine, other faculties at the University of Bergen, or by other universities or university colleges in Norway or abroad. Where academic discipline-oriented or research area-related courses have been organised at the Faculty, these are expected to be included in the training component.  Participation in a research course that is not credited using ECTS standards may be approved on application, for calculating credits. Approval requires that documentation for the course is submitted (schedule, course description and syllabus), in addition to proof that the candidate has completed and passed the course.  In addition, the guidelines states that if the candidate travels to a research institution abroad to learn techniques, conduct experiments or engage in other academic activities, the stay abroad may be approved as part of the training component's elective part. After returning, a report about the stay must be submitted (signed by the candidate and supervisor) in addition to a confirmation from the host institution regarding the duration of the stay and its content | Any educational activity or experience that is relevant for the individual study plan for the PhD student can be approved by the Departmental Director of Doctoral Education for accreditation. | 0 |  |  |  |  |  |  |
| #63  QD  8.1 | There ought to be procedures for regular review and updating of the structure, function and quality of PhD programmes, including both supervisor and candidate feedback. | Annual Review and Enhancement (ARE) draws upon a range of qualitative and quantitative evidence including External Examiners reports and information received from Professional, Statutory and Regulatory Bodies to enable forward planning based on focused reflection and evaluation of data and relevant feedback, with a view to continuous enhancement of our taught and research provision. The outcomes of the ARE process informs planning processes at School level and provides the College and the University with appropriate information for oversight of the quality assurance and enhancement of research degree programmes. | Regular national PRES (Postgraduate Research Experience Survey) <https://www.heacademy.ac.uk/institutions/surveys/postgraduate-research-experience-survey> for candidate feedback. University has full PGR programme review every 5 years involving external assessors. In addition there is a shorter annual review to the Faculty PGR Committee. | **The Programme Board** is a main forum to review and update the structure, function and quality of PhD programme. Both supervisors and candidates have representatives in the board. In addition, individuals may submit matters to the board.  **The annual supervisor seminars** are a second forum to review, discuss and propose changes to the PhD programme. Although the supervisor seminars don’t have authority to make decision, propositions may be presented to the Programme Board  **The annual progress report and midway evaluations** may act as a third way for the individual candidate or supervisor to review the PhD programme and propose updates. | There are multiple points of evaluation and quality control, as described in BS3.3, including annual review and halftime review procedures. | 0 |  |  |  |  |  |  |
| #64  QD  8.2 | Representatives of the PhD candidates ought to interact with the leadership of the graduate school regarding the running of the graduate school. Candidate organisations ought to be encouraged and facilitated. | The Students’ Union is a democratic organisation with a membership of over 30,000 students. In February of every year, students are elected as representatives for the following academic year. The Vice President Postgraduate Students represents 12,500 postgraduates at Cardiff University on academic policies that affect taught or research postgraduate students, raising welfare concerns and reviewing the support structures that are currently in place, or simply helping to promote and expand on the vibrant postgraduate community at Cardiff. PGR Reps play an integral part in improving the academic standards and quality of teaching at Cardiff University. They help shape the student experience. In total there are over 1000 Academic Reps in all 25 schools. <https://www.cardiffstudents.com/about-cusu/postgrad/postgrad-officer/> | See BR8.1 and BR8.2 explaining about structure of graduate school and University. Interaction with the Institute and Faculty are therefore important.  The IIB PGR Committee has PhD candidates, academic and administrative staff. A postgrad from this committee sits on the Faculty PGR Committee which is to relevant running the graduate school and also the IIB Steering Group (relevant to IIB running PhD programmes). | PhD candidates interact with the leadership of the graduate school in the following ways:  **Participation in the Programme Board and Board for extended Research Management**: PhD candidates are permanent members with voting rights in both the Programme Board and Board for extended Research Management.  [**EUREKA**](http://www.uib.no/en/node/66335)**, the student organisation** for The Medical Student Research Programme at The Faculty of Medicine (NB! Website only in Norwegian) | Student representatives are included in all KI Boards (Education, Doctoral Education & Research), in Departmental Steering Boards, in Departmental Admission Boards, and in sub-­‐groups or other under organisations of these (e.g. dissertation committee, recruitment committee).  Student influence is thus encouraged at every level and is taken seriously. | (0) |  |  |  |  |  |  |
| #65  QD  8.3 | PhD candidates ought to have rights and duties commensurate with the value (to the institution) of the research performed. | The University recognises that supporting the learning of others provides a valuable opportunity for research students to develop their professional skills. As part of their overall development, research students may seek teaching opportunities: to work as tutors or demonstrators; to co-supervise undergraduate projects; and to assess students' work. The Code of Practice for PGR Teaching outlines the University’s principles in relation to the recruitment, selection, training, monitoring and remuneration of research students engaged in teaching activities. | See Section 7 in <https://www.liverpool.ac.uk/media/livacuk/tqsd/code-of-practice-on-assessment/appendix-2-PGR-CoP.pdf> . See also LDC handbook chapters 3 and 4 <https://www.liverpool.ac.uk/media/livacuk/pgrstudentteam/LDC,PGR,Handbook,2017-18.PDF>  Stipend is typically at rate set by RCUK and **for 2017/18 is £14,553 outside London (tax free,** no NI or pension contribution) <http://www.rcuk.ac.uk/media/news/170104/> Organisations are free to pay higher rates. | The rights and duties of PhD candidates are specified in the PhD regulation and in the AGREEMENT RELATING TO INCLUSION IN THE ORGANISED TRAINING OF RESEARCHERS AT THE UNIVERSITY OF BERGEN.  The Faculty of Medicine does not offer any kind of scholarship or other financial benefits beyond research fellows. For UiB research fellows, the general pay and working conditions are controlled by the Basic Collective Agreement for the Civil Service. The Faculty has differentiated salary for the UiB research fellows:   1. New candidates begin at pay grade 50, NOK 429.700-, with yearly increases in pay 2. Candidates with completed medical internship or dentists with one year practice work begin at pay grade 52, NOK 443.900-, with yearly increases in pay 3. Candidates with completed medical specialist training is placed in pay grade 58, NOK 491.400-, with no further increases in pay   Other aspects of the employment relationship are regulated by, among other things, the Act relating to Universities and Colleges (uhl), the Civil Service Act (tjm1), the Working Environment Act (aml), the National Insurance Act, the Act relating to the Norwegian Public Service Pension Fund, the Act relating to Age Limits, the Act relating to Public Services Disputes, the Basic Agreement for the Civil Service including the adjustment agreement applicable at UiB, the personnel regulations and any special agreements that may be relevant to the position. | This is in accordance with KI philosophy. | 2 |  |  |  |  |  |  |
| #66  QD  8.4 | There ought to be an appeal mechanism allowing PhD candidates to dispute decisions concerning their programmes and thesis assessment. | Candidates who are not recommended by the Examining Board for the award of Doctor may appeal against the decision reached, in accordance with the Academic Appeals Procedure at Cardiff University. | See <https://www.liverpool.ac.uk/media/livacuk/tqsd/code-of-practice-on-assessment/appendix-10-PGR-CoP.pdf> | The appeal mechanisms for PhD candidates to dispute general decisions **concerning their PhD programmes** are regulated through the PhD regulation’s §§16-18.The PhD regulation’s §§ 16-18 are in accordance with the Public Administration Act § § 28. Appeals can be presented to the proper authority which will vary depending on the matter.  The appeal mechanisms for PhD candidates concerning the **thesis assessment** specifically are regulated through the PhD regulation’s §18, which states that a rejection of a thesis, trial lecture or defence may be appealed in accordance with the provisions of § 28 and following of the Public Administration Act. The appeal must be sent to the Faculty, and must detail the grounds on which the rejection is being appealed. After the case has been presented to the assessment committee, the Faculty may set aside or amend the decision if it finds the appeal to have been substantiated. If the Faculty does not allow the appeal, the appeal is sent on to the Central Appeals Committee for a decision. The Appeals Committee may test all aspects of the appealed decision. If the subsidiary body or the appeals body finds reason to do so, a committee or a number of individuals may be appointed to evaluate the assessment that has been made and the criteria the assessment was based on, or to perform a new or supplementary expert assessment. | In accordance with the Governmental Higher Education Ordinance the following decisions can be disputed by PhD students:  Accreditation of ECTS-­‐denoted activities  Formal mandatory activities with PhD  Withdrawal of support by the Department  Denial of obtaining examination certificate  Non-­‐approval of a thesis defence cannot be disputed but re-­‐examination at a later date can be arranged in agreement with all parties. | 0 |  |  |  |  |  |  |
| #67  QD  8.5 | Confidential candidate counselling concerning e.g. the PhD programme, supervision, as well as personal matters ought to be offered by the graduate. | Student support and wellbeing services at Cardiff University deliver a comprehensive range of services dedicated to helping our PGRs make the most of student life. Student Support Centres are provided on both the Cathays and Heath Park Campuses where free, impartial, non-judgemental and confidential advice on a wide range of matters is provided (e.g., Money, careers, employability, counselling and wellbeing, disability and dyslexia, international student support, student mentor scheme, equality, diversity and inclusion, practising your religion and day care centre. | IIB PGR Pastoral Group can sign-post candidates but are not trained counsellors. The University Student Services offers counselling and is completely confidential (hence numbers not known with any confidence). See <https://www.liverpool.ac.uk/studentsupport/counselling/> . Counselling can also be available via the candidate’s general practitioner (through National Health Service). | **PhD coordinators at the departments:** Each department have at least one PhD coordinator who acts as a primary point of contact for all PhD candidates affiliated with that specific department. The PhD coordinators are considered independent of the doctoral school leadership and supervisors. As PhD coordinators are located at the departments, close to the research environments, they are easily available for both candidates and supervisors all year.  **Head of Research at the Department:** Each department also have a Head of Research (or equivalent position). The Head of Research is responsible for matters concerning research and the doctoral education at the departmental level. The Head of Research is independent of the doctoral school leadership and supervisors.  Although the Faculty holds no statistics of how many candidates use such counselling facilities, the departments report that they handle a few matters each year. | Every student has the following options for formal counselling:  Departmental Director of Education: First-­‐line support.  Departmental Head: Preferred choice in case of involvement of the Departmental Director of Doctoral Education in the situation.  Central Director of Doctoral Education: Preferred choice in case of involvement of Dept Head or Dept Director in the situation.  External Mentor: Defined at admission as a trustworthy confidant.  Doctoral Ombudsman: Employed by the Student Union (including both undergraduate and graduate students) to mediate and support all PhD students (confidentiality).  Dean of Doctoral Education: has the ultimate responsibility delegated by the KI President, and can be directly involved in the process if the student wishes.  Healthcare professionals: In case of the need for counselling this service is free for students (confidentiality). | 2 |  |  |  |  |  |  |
| #68  QD  8.6 | Graduate schools could consider having a thesis committee for each PhD candidate that monitors the progress of the PhD candidate through meetings with the PhD candidate and the supervisors. | The progress of every research student is formally reviewed on an annual basis up until the successful submission of the thesis. The first Formal Progress Review is normally completed within 9 months of the student first registering, and at 12-monthly intervals thereafter (so normally completed within 9, 21, 33 months). At the annual review a the student meets with the Review Panel (3 members not including supervisor) and progress/pathway to completion is assessed by running a mini-viva | The University has a formal arrangement of independent progress assessment panels (IPAP) to meet candidate and monitor progress to PhD. Assessors provide a report to Institute on student’s progress annually. Supervisors can provide written information to assessors but cannot attend the meeting.  See <https://www.liverpool.ac.uk/media/livacuk/tqsd/code-of-practice-on-assessment/appendix-3-PGR-CoP.pdf>  See also BR4.7 | As stated in the PhD regulation’s §6.1, all candidates who are admitted to the PhD programme at The Faculty of Medicine, must have at least two supervisors. In addition to this all candidates must undergo a midway assessment. For this purpose, a dedicated midway evaluation committee is appointed to assess the progress of the candidate and to give academic input within the candidate's field and/or related fields. Thus, it is not considered necessary to have a thesis committee that monitors the progress of the candidate. | It is recommended that all three or some of the halftime review committee be included in the final thesis defence examination committee, in order to contextualise better the progress of a give student.  Appointment of a defined thesis committee has been discussed, but is considered to be both unnecessary given the current structure, and unmanageable considering the volume of PhD students within KI. | 1 |  |  |  |  |  |  |

1. *Applicants with UiB fellowships, go through this process as part of the application for the UiB fellowship. Such candidates do not repeat this process when applying for admission to the PhD programme.* [↑](#footnote-ref-1)
2. *Statutory leaves are not included in the statistics regarding PhD period for candidates.* [↑](#footnote-ref-2)
3. *Statutory leaves are included in the statistics regarding PhD period for candidates.* [↑](#footnote-ref-3)
